# Quantifying Cellular Uptake of Nanoplastics *In Vitro*: A Workflow for Fluorescently Labeled Nanosized Polystyrene Particles

**DOI:** 10.1021/acsomega.6c02589

**Published:** 2026-07-27

**Authors:** Markus J. Kirchner, Francesca Bennet, Adriaan J. A. Duijndam, Florian Meirer, Christian Laforsch, Alexander Roloff

**Affiliations:** 1 Department of Chemical and Product Safety, 27652 German Federal Institute for Risk Assessment (BfR), Max-Dohrn-Str. 8−10, 10589 Berlin, Germany; 2 Animal Ecology I, Bayreuth Center of Ecology and Environmental Research (BayCEER), University of Bayreuth, Universitätsstr. 30, 95447 Bayreuth, Germany; 3 Inorganic Chemistry and Catalysis, Institute for Sustainable and Circular Chemistry, 8125 Utrecht University, Universiteitsweg 99, 3584CG Utrecht, The Netherlands

## Abstract

The accumulation of micro- and nanoplastics (MNPs) in the environment increasingly entails human exposure to this class of highly diverse polymer particles. To evaluate potential risks to human health, hazard assessments commonly involve *in vitro* testing using human-derived cell lines to establish dose–response relationships for MNPs. However, the reliable quantification of actual particle uptake in cells is particularly challenging for nanoscale materials (particles less than 1 μm in size). We present a workflow for the characterization and quantification of the *in vitro* uptake of fluorescently labeled polystyrene (PS) nanoparticles in human-derived A549 lung epithelial cells as a relevant model for inhalation exposure. This study employs well-characterized PS nanobeads with a mean diameter of 180 nm. The semiquantitative characterization of uptake by confocal fluorescence microscopy and flow cytometry is complemented by two orthogonal quantification methods based on automated fluorescence imaging microscopy and online pyrolysis gas chromatography mass spectrometry (Py-GC-MS), the latter also rendering unlabeled MNPs accessible. Applying the developed workflow, we confirm that PS nanoplastics are taken up in significant amounts by A549 cells. Analysis by flow cytometry revealed that almost all cells take up particles. Applying an automated high-throughput fluorescence microscopy platform, we determined a dose-dependent uptake resulting in an average accumulation of 1100 ± 450 particles per cell (mean ± SD) when incubated for 24 h with the highest tested dose of 31 μg·cm^–2^. These findings were confirmed by Py-GC-MS, a method used for MNP quantification in human cells *in vitro* for the first time, yielding on average 3.2 ± 0.6 pg PS (950 ± 200 particles) per cell. The workflow described in this study facilitates characterization and quantification of cellular MNP uptake *in vitro,* allowing for the calculation of average particle counts and polymer mass contents of nanosized plastic particles per cell.

## Graphical abstract

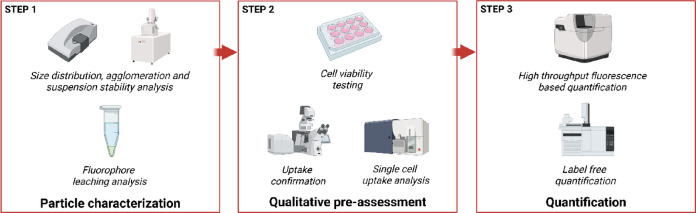

## 1 Introduction

Due to their pervasive presence, micro- and nanoplastic particles (MNPs) are of rising interest both in a regulatory and societal context.^1^ In order to properly assess whether MNPs may pose risks to human health, knowledge of the exposure and associated hazards for this class of highly diverse and ubiquitous environmental contaminants is needed. While significant efforts have been made to explore oral uptake of MNPs,^2^ exposure via the inhalation route is less understood.^1,3,4^ However, polymer particles have been detected in both indoor and outdoor air^5–9^ as well as in lung tissue,^10,11^ the latter indicating that polymer particles of certain sizes can accumulate in the human respiratory tract. This is supported by modeling approaches suggesting that smaller particles (<1 μm) could indeed reach the distal lungs, including the alveolar system, upon inhalation.^12^

Dose–response relationships for the biological effects established in *in vitro* assays are needed in order to assess hazards associated with MNP exposure.^13^ Despite the critical role of both MNP-cell-interactions and cellular internalization (the sum of these processes is referred to as “uptake” in this manuscript) as important initial steps preceding biological responses, many studies lack a robust quantification of MNP uptake.^14^ Importantly, biological effects should not only be related to externally applied doses derived from particle deposition but also to the actual mass and area-specific dose of MNPs that cells take up, as these parameters are key determinants of many toxicological responses. At the same time, the number of particles provides complementary information, because exposures with similar nominal mass can correspond to very different particle counts and thus different frequencies of particle–cell interaction events, especially when size distributions or aggregation states differ. Hence, quantifying both polymer mass and particle number at the cellular level is necessary in order to enable a more nuanced interpretation of dose–response relationships across different MNP size regimes and material types.

To date, quantification approaches for MNPs in cellular contexts typically rely on the use of surrogates such as metal or fluorophore labels. For example, cellular uptake of MNPs with incorporated gold nanoparticles has been quantified with single-cell inductively coupled plasma mass spectrometry. The incorporated gold particles also allowed for qualitative confirmation of uptake by transmission electron microscopy.^15^ Others have used fluorophores embedded in the polymer particles to analyze cellular uptake *in vitro* using confocal fluorescence microscopy.^16–18^ This technique also provides information on whether MNPs are located on the inside or outside of cells. However, without proper calibration this method is limited to relative quantification of dose-dependent increases in fluorescence intensity. Another study reported on a high-throughput fluorescence microscopy method to examine uptake of fluorescently labeled PS MNPs by assuming different quantum yields of internalized particles compared to free PS particles.^19^ Similarly, without calibration, this approach cannot provide absolute values for particle uptake. Importantly, possible fluorophore leaching needs to be considered, since accumulation in hydrophobic cellular compartments such as lipid droplets might imitate particle-like shapes.^20^ Flow cytometry has been used for quantifying relative MNP uptake by measuring changes in the fluorescence intensities of individual cells. However, most studies either did not provide numbers for the average particle uptake per cell (particularly for smaller submicron particles),^18,21,22^ or derived these values from additional fluorescence microscopy imaging analyses from a comparatively small number of cells.^23^ Moreover, while flow cytometry provides high-throughput readouts, both the cytometry and microscopy measurements ultimately rely on the same fluorescence-based readout, making this approach intrinsically affected by fluorophore loading, quenching, dye leakage and autofluorescence. Implementing another independent surrogate for quantification, or, ideally, quantifying the polymer itself would help validating single-fluorophore based uptake data and strengthen the significance of such results.

Regardless of labeling, particle size often is a limiting factor for detection—especially for nanoscale particles. For example, confocal fluorescence microscopy combined with differential intensity contrast microscopy was shown to be suitable for detecting and quantifying uptake of unlabeled 3–10 μm PS beads. To circumvent the use of labeled particles, filamentous actin was fluorescently labeled to distinguish between internalized and membrane-attached microplastic particles (MPs). Only MPs fully surrounded by fluorescently labeled actin were considered to have been internalized. However, while particles below 1 μm can still be detected, accurate size determination becomes increasingly difficult below the optical diffraction limit.^24–27^

An approach that does not rely on the detection of chemical labels, which are typically absent on environmental MNPs, is online pyrolysis-gas chromatography–mass spectrometry (Py-GC-MS). Py-GC-MS relies on the specific mass spectrometry-based quantification of characteristic polymer fragments formed during pyrolysis, thereby providing analysis of polymer masses in complex samples. Py-GC-MS has been widely used to characterize polymers and environmental microplastics.^28–30^ Recently, Nagano and co-workers applied Py-GC-MS to quantify ingested polystyrene (PS) microspheres in individual *Daphnia magna*, demonstrating that this technique can be used to assess microplastic burdens in small aquatic organisms.^31^ However, that study did not address nanoplastics nor polymer quantification in mammalian cell systems. Extending pyrolysis-GC-MS to complex cellular matrices introduces challenges, including copyrolysis of cellular components, matrix-dependent recoveries and the need for rigorous calibration strategies. At the same time, Py-GC-MS offers a key conceptual advantage over fluorescence-based approaches because it targets the polymer itself and can therefore, in principle, be transferred to nonfluorescent and, hence, environmentally realistic particles after polymer- and matrix-specific calibration and validation. Recent work has further highlighted the value of standardized Py-GC-MS workflows for detecting nanoplastics and microplastics in complex environmental and biological matrices.^32–34^

In summary, there currently is no established workflow for accurately quantifying the *in vitro* uptake of nanoplastic particles in human cells. Therefore, we aimed to integrate state-of-the-art analytical techniques, including spectroscopic and mass-based methods, to reliably characterize and quantify the uptake of nanoscale PS beads *in vitro* in human-derived A549 epithelial lung cells (Scheme 1). A549 cells were selected as a robust and widely used human alveolar epithelial model that allows comparison with a broad body of literature on inhalation toxicology and nanoparticle uptake, although this transformed cell line does not fully recapitulate the physiological complexity of the airway barrier *in vivo* due to, e.g., the lack of mucociliary clearance.^35^ The approach developed here addresses an important gap between particle imaging techniques and bulk polymer analysis. As a proof-of-concept, all experiments were carried out with spherical (*d* = 180 nm) fluorescently labeled PS nanoparticles (PS-NPs). The choice of material is justified by the relevance of the polymer for human respiratory exposure^10^ as well as its commercial availability with standardized size, shape and fluorophore content. Although these particles do not reflect the typical characteristics of MNPs found in the environment,^8,36,37^ this well-defined model system provides the degree of physicochemical control required for cross-method verification and benchmarking. The present study should therefore be understood as a methodological proof-of-concept rather than an investigation aimed at assessing cellular exposure toward environmentally aged, irregularly shaped, and chemically heterogeneous MNPs. Nevertheless, the workflow is intended to support future transfer to more complex particle systems, particularly where polymer-specific quantification by Py-GC-MS can complement fluorescence-based single-cell readouts.

**Scheme 1:**
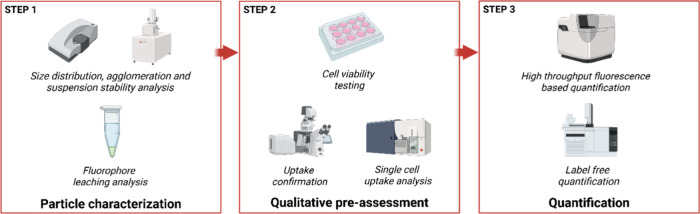
Workflow for Characterization and Quantification of Nanoplastic Uptake *In Vitro* ^a^

a (1) Characterization of particle size distribution, agglomeration and fluorophore-leaching. (2) Qualitative pre-assessment to establish a dose range and confirm particle uptake. (3) Quantification and confirmation of particle uptake employing fluorescence-based and label-free analytical methods (images were created in BioRender by M. Kirchner, https://BioRender.com/b2psjzq). The workflow consists of three steps to characterize and quantify cellular uptake of nanoplastics *in vitro*. In Step 1, particles are initially characterized by (i) dynamic light scattering to determine size and potential agglomeration in relevant media, (ii) scanning electron microscopy to confirm particle size and shape, and (iii) evaluation of possible fluorophore leaching in a biphasic octanol–water system as well as in cell culture medium via fluorescence spectroscopy. The qualitative pre-assessment (Step 2) includes a cell viability assay to establish a dose regime for the *in vitro* testing of MNP uptake in cells without significantly affecting cell viability. Particle uptake in A549 lung cells is characterized qualitatively via 3D fluorescence imaging using confocal microscopy. Subsequently, flow cytometry analysis is applied to determine the percentage of cells that take up particles, revealing different uptake efficiencies between individual cells or cell subpopulations. Quantification of particle uptake is accomplished in Step 3 with automated high-throughput fluorescence imaging microscopy measuring particle-associated fluorescence intensities within the area of stained cellular structures. Results of fluorescence imaging microscopy are compared with label-free quantification by Py-GC-MS, revealing average polymer masses per cell.

## 2 Materials and Methods

### 2.1 Materials and Chemicals

Fluorescently labeled spherical PS particles with a mean diameter of 180 nm and a polydispersity index (PDI) of 0.021 were purchased from Kisker Biotech (Steinfurt, Germany; stock concentration 10 mg·mL^–1^). According to the supplier, excitation and emission wavelengths for the incorporated fluorophore are comparable to those of fluorescein isothiocyanate (FITC): *λ_ex./em._* = 470–490/520 nm. The particle size was confirmed by dynamic light scattering (DLS) and scanning electron microscopy (SEM). The stability of the aqueous particle suspensions was verified by zeta potential measurements. The agglomeration behavior of the particles in cell culture medium was investigated over 24 h by DLS, and leaching of the fluorophore was assessed in an octanol/water system and in cell culture medium (see Section 2.2).

All relevant cell cultivation media and supplements (antibiotics, amino acids, enzymes, fetal calf serum) were purchased from PAN Biotech (Aidenbach, Germany). 96-well plates, 24-well plates and T75 flasks were acquired from TPP Techno Plastic Products AG (Trasadingen, Switzerland). V-bottom 96-well plates were obtained from Greiner Bio-One GmbH (Frickenhausen, Germany). Formaldehyde solution (4%, Rotihistofix), 3-(4,5-dimethylthiazolyl-2)-2,5-diphenyl-2*H*-tetrazoliumbromid (MTT), Triton-X 100 and dichloromethane were purchased from Carl Roth (Karlsruhe, Germany). CD58-APC monoclonal antibody, Hoechst 33342 and Alexa Fluor 647 Phalloidin were acquired from Thermo Fisher Scientific (Life Technologies GmbH, Darmstadt, Germany). Mounting medium and 8-well chambers were purchased from ibidi GmbH (Gräfeling, Germany). PS standard for Py-GC-MS was obtained from Fluka Honeywell (Charlotte, NC, USA) and 4F-polystyrene from Polymer Source (Dorval, Canada). Dimethyl sulfoxide (DMSO) was purchased from Honeywell Riedel-de Haën (Seelze, Deutschland). Milli-Q water was produced using a Milli Reference system (Merck KGaA, Darmstadt, Germany), aliquoted in a 50 mL glass bottle (DURAN Life Science Holding GmbH, Wertheim, Germany) and subsequently autoclaved twice before use.

### 2.2 Methods for Material Characterization

Hydrodynamic diameter and zeta potential of the fluorescently labeled spherical PS particles were measured with a Zetasizer Nano ZS (Malvern Panalytical GmbH, Kassel, Germany). The PS-NP suspensions were analyzed in disposable PS cuvettes (500 μL) at room temperature at concentrations of 100 μg·mL^–1^ in Milli-Q water. Results for hydrodynamic diameters are given as intensity-based size distribution histograms, Z-average diameter and PDI. The zeta potential was determined in reusable quartz cuvettes (1000 μL) employing the universal "Dip" Cell Kit (Malvern Panalytical GmbH, Kassel, Germany). Mean values and standard deviations (SDs) of at least three independent samples were calculated for all measured parameters.

To investigate possible agglomeration in the cell culture medium, DLS measurements were conducted with a 100 μg solution of PS-NPs in Dulbecco’s modified eagle medium (DMEM) after 0, 4, 8, and 24 h. At least two samples were analyzed for each time point.

For scanning electron microscopy (SEM) measurements, a droplet (ca. 10 μL) of PS-NP suspension was drop-cast onto a 10 mm × 10 mm square Si wafer (single side polished, B-doped, 525 ± 20 μm thickness, Siegert Wafer) which was subsequently placed on a heating plate set to 50 °C inside a laminar flow cabinet to ensure drying of the droplet in a clean environment. The wafers were stuck onto alumina SEM-stubs (12.7 mm slotted head, 3.18 mm pin, Ted Pella) with carbon tape (Electron Microscopy Sciences). A Pt-coating of 7 nm was applied using a sputter coater (Cressington 208HR) equipped with a thickness controller (Cressington MTM20). SEM measurement were performed using a Helios Nanolab G3 instrument (Thermo Fisher Scientific) with a beam current of 25 pA and an acceleration voltage of 2 kV, using either an Everhart-Thornley Detector or a Through-Lens Detector, depending on the magnification. Four images (750 particles in total) were analyzed for particle diameter using ImageJ software (open source).

To assess possible fluorophore leaching from the particles, PS-NP suspensions (100 μg·mL^–1^, corresponding to 31 μg·cm^–2^ in experiments involving exposed cells) in Milli-Q water were incubated with equal amounts of *n*-octanol and vortexed for 30 s. After phase separation, the fluorescence intensities (*λ_ex./em._* = 490/520 nm) of both phases were measured individually using a plate reader (BioTek Synergy Neo2, Agilent, Waldbronn, Germany). The biphasic mixtures were further agitated (300 rpm) for 24 h at 37 °C in the dark and analyzed under identical conditions. Additionally, for both time points, the separated phases were centrifuged for 50 min at 19,000 rcf to spin down PS-NPs and the supernatants were analyzed as described above. Furthermore, fluorescent PS-NPs (100 μg·mL^–1^) were incubated in complete cell culture medium at 37 °C. Immediately after preparation and again after 24 h incubation, samples were centrifuged for 50 min at 19,000 rcf to pellet the particles. In parallel, medium without particles was processed as a control. The fluorescence of the particle-free supernatants and the corresponding medium controls was measured as described above. To account for background fluorescence from phenol red in the cell culture medium and potential inter-day variability, all fluorescence signals were normalized to the respective medium control analyzed in the same run. Fluorophore leaching experiments were performed in three technical replicates each, with results reported as mean values (relative SDs were < 7%).

### 2.3 Cultivation of the Lung Cell Models and Cell Viability Testing

A549 human lung carcinoma cells derived from a 58-year-old Caucasian male^38^ are an often-used lung model in toxicological research. The human alveolar type II epithelial cell line, purchased from the German Collection of Microorganisms and Cell Cultures GmbH (Leibniz Institute DSMZ, Deutsche Sammlung von Mikroorganismen und Zellkulturen), was grown in DMEM supplemented with 10% (*V*/*V*) fetal calf serum, 2 mM l-glutamine, 100 U·mL^–1^ penicillin and 100 mg·mL^–1^ streptomycin in a humidified incubator at 5% CO_2_ and 37 °C. Cells were passaged twice weekly and medium was replaced the day after passage. Passages 4 to 20 were used in the following experiments. Cells were regularly tested negative for mycoplasma.

6.25 × 10^5^ cells·cm^–2^ were allowed to adhere for 24 h before the start of experiments. This seeding density was chosen to establish a rather dense lung epithelial monolayer, providing a physiologically more relevant *in vitro* lung model to study nanoparticle uptake.^39^ Cell viability testing and automated fluorescence imaging microscopy were performed in 96-well plates, while experiments involving flow cytometry and pyrolysis-GC-MS were conducted in 24-well plates. Cells for confocal microscopy were seeded in 8-well chamber slides.

Cell viability testing was performed by incubating the cells with 31, 3.1, and 0.31 μg·cm^–2^ fluorescent PS particles in DMEM (containing 10% Milli-Q water), corresponding to 100, 10, and 1 μg·mL^–1^. Medium containing 10% Milli-Q water or 1% Triton-X 100 (added 30 min before the end of an assay) was used as negative and positive controls, respectively. After incubation for 24 h the cells where washed three times with PBS and then incubated for 2 h at 37 °C with 0.5 mg·mL^–1^ MTT solution in medium in the dark. After removing the supernatants, 100 μL DMSO were added, and the plate was shaken in an orbital shaker (450 min^–1^) for 15 min to dissolve the formazan crystals. Absorption was measured at 570 nm and corrected for the background absorption at 630 nm using a multimode microplate reader (BioTek Synergy Neo2, Agilent, Waldbronn, Germany). Mean values and SDs were calculated from three technical replicates of three independent biological replicates representing cells with different passage numbers.

### 2.4 Confocal Fluorescence Microscopy for Qualitative Confirmation of Particle Uptake

A549 cells were seeded as described above and subsequently incubated for 24 h with the highest previously assessed and nontoxic dose of 31 μg·cm^–2^ fluorescently labeled PS particles in DMEM (see Section 3.2). After incubation, cells were fixed for 20 min at 37 °C using 4% formaldehyde solution, permeabilized for 20 min at room temperature with 0.2% Triton-X 100 solution and stained for 30 min at 37 °C in the dark with Alexa Fluor 647 Phalloidin (actin skeleton) and Hoechst 33342 (DNA/nuclei). After staining and washing with PBS (3×) and Milli-Q water, mounting medium was added to the well-chambers. Measurements were performed on a confocal microscope (LSM 700, Carl Zeiss Jena GmbH, Jena, Germany) using the ZEN 2012 black edition software (Carl Zeiss Jena GmbH) for instrument control. Microscopy images were taken with consistent laser light transmissions (0.8–2.0%) using the z-stack option with at least 30% overlap of each image with the following one resulting in approximately 20 to 24 images per z-stack covering an approximate distance of 11 to 14 μm. Four measurements were averaged for each image. The confocal pinhole was set to a range of 1.22–1.79 Airy units to provide sufficient z-sectioning. Each image series comprised frames with a resolution of 512 × 512 pixels, a pixel size of 400 nm, and a pixel dwell time of 2.55 μs. The start and stop of the z-stack were visually defined for every replicate according to the Alexa Fluor 647 Phalloidin stain. Excitation of fluorophores was performed with individual lasers (Hoechst 33342: 405 nm, fluorescently labeled PS-NPs: 488 nm, Alexa Fluor 647 Phalloidin: 639 nm) in best signal mode allowing for consecutive excitation of the fluorophores and read-out of the relevant emissions in each channel (photomultiplier settings: Hoechst 33342: 400–490 nm, fluorescently labeled PS-NPs: 400–630 nm, Alexa Fluor 647 Phalloidin: 400–700 nm).

ZEN 2012 blue edition software (Carl Zeiss Jena GmbH) was used for data analysis. Cell-associated regions within each image were defined by outlining all Alexa Fluor 647 Phalloidin-stained compartments (actin). The emission of the fluorescently labeled PS-NPs originating from within the defined intracellular areas was quantified. Subsequently, the area-specific fluorescence intensities of every image were summarized for each z-stack.

### 2.5 Flow Cytometry to Assess Particle Uptake Efficiency

We included immunostaining of cells prior to analysis to exclude contributions from noncell-based structures to the PS-NP-associated fluorescence signal in the FITC channel. Thus, A549 cells were incubated with fluorescently labeled CD58 antibodies, a marker that is expressed ubiquitously even after stimulation with e.g. interferons.^40^

To minimize autofluorescence, the voltages for the photomultiplier were adjusted to result in background fluorescence intensities in the FITC channel of lower than 10^3^ r.f.u for more than 99% of untreated cells. Events above this threshold were considered positive in terms of particle uptake.

A549 cells were seeded as described above and subsequently incubated for 24 h with the highest previously assessed and nontoxic dose (see Section 3.2) of 31 μg·cm^–2^ fluorescently labeled PS particles in DMEM. Cells were washed three times with PBS, trypsinated for 5 min (0.5% trypsin/0.02% EDTA in PBS), diluted with medium and transferred into a V-bottom 96-well plate. The cells were centrifuged at 400 rcf for 6 min and washed twice with 1% FCS in PBS before 20 min incubation at room temperature with a CD58-APC antibody (80-fold dilution of the stock solution) in PBS. Suspensions of four wells were combined in one flow cytometer tube. Analysis was performed with a BD FACSAria III flow cytometer using the BD Diva7.0 Software (BD Biosciences, San Jose, USA) for instrument control. The gating strategy for analysis is described in the Supporting Information (Figure S1A). Data was analyzed using FlowJo software (BD Biosciences, San Jose, USA).

### 2.6 Automated Fluorescence Imaging Microscopy for High-Throughput Quantification of Particle Uptake

A549 cells were seeded as described above and subsequently incubated for 24 h with 31, 15.5, 7.8, and 3.9 μg·cm^–2^ fluorescently labeled PS particles in DMEM to assess dose dependency of the uptake. After incubation the cells were fixated, permeabilized and stained as described in Section 2.4. Automated fluorescence imaging microscopy was performed on a Cell Discoverer 7 instrument (Carl Zeiss Microscopy GmbH, Jena, Germany) using the ZEN 3.1 blue edition software for instrument control and data analysis (Carl Zeiss Microscopy GmbH). The device was equipped with a 90 HE LED filter unit allowing for excitation at wavelengths and bandwidths of 385 nm/30 nm (Hoechst 33342), 469 nm/38 nm (fluorescently labeled PS-NPs) and 631 nm/33 nm (Alexa Fluor 647 Phalloidin), applying an LED intensity of 50% in all experiments. For read-out, emission filters with wavelengths and bandwidths of 425 nm/30 nm (Hoechst 33342), 514 nm/30 nm (fluorescently labeled PS-NPs) and 709 nm/100 nm (Alexa Fluor 647 Phalloidin) were used. Five images of 900 μm × 650 μm size were acquired for each well, with one automatically positioned in the center of the well and four equally positioned around it. These images were then averaged as one technical replicate. Hoechst 33342 (nuclei stain) was used as the reference stain for autofocus on the *z*-axis.

A 7-point calibration was prepared from a stock suspension of fluorescently labeled PS-NPs in Milli-Q water. Aliquots of diluted particle suspensions (1.56 to 18.75 pg·μm^–2^) were added to a 96-well plate and centrifuged for 60 min at 2,773 rcf to ensure sedimentation of the particles. For calibration, the fluorescence intensities of the particles in the entire well areas were recorded with 35 ms exposure time (this signal was also used as the reference stain for autofocus on the *z*-axis) and subsequently translated into area-specific fluorescence intensities.

For data analysis, an intensity threshold of 1100–1300 relative fluorescence units (r.f.u.) was defined in the analysis section of the software for the actin stain Alexa Fluor 647 Phalloidin (25 ms exposure time) in order to distinguish intracellular regions from other areas (representative images shown in Figure S2). The threshold was adjusted for every passage depending on the staining efficiency; the software therefore allows for the outlining of all cellular sections in the images. The same approach was used to outline all nuclei (Hoechst 33342 nuclei stain, 10 ms exposure time, threshold to distinguish nuclei form non-DNA containing regions: >2200–2500 r.f.u.), with special attention to the separation of adjacent cell nuclei. The adjusted values were visually checked for several randomly chosen images of the batch and then applied to all measurements of the passage for automated processing. Emissions of the fluorescent PS-NPs (35 ms exposure time) colocalized with actin-stained regions were recorded and corrected by subtracting average results for cells not incubated with PS-NPs to account for possible autofluorescence. Results are presented as number of nuclei, cellular area of the Alexa-647 stain and area-specific fluorescence intensities of cell-associated PS-NPs for each image, respectively.

To calculate the average particle number per cell, the area-specific fluorescence intensities can be translated into polymer masses per area by external calibration. Furthermore, the ratio of this cell-associated area-specific polymer mass and the number of nuclei represents the polymer mass per cell. Assuming an average mass of 3.33 fg for a 180 nm spherical PS particle with a density of 1.09 g·cm^–3^ ^41^ an average particle number per cell was calculated (see Section S3 for details). The reported results are based on the assumptions that (i) the quantum yield of the fluorophore associated with the PS-NPs in the calibration suspension is the same as inside the actin-positive regions of cell samples, as well as (ii) light scattering in both matrices is similar.

### 2.7 Pyrolysis Gas Chromatography Mass Spectrometry (Py-GC-MS) for Confirmation of Particle Uptake via Quantification of the Cell-Associated Polymer Mass

Py-GC-MS is based on the concept of thermal degradation of polymers into shorter fragments that are amenable to separation by gas chromatography and subsequent quantification by mass spectrometry. While this technique is gaining popularity for quantifying polymer contents in biological tissues such as blood, placenta and brains,^30,32,42,43^ reports for *in vitro* cell culture applications are lacking so far.

During pyrolysis, PS mainly degrades into styrene monomers, dimers and trimers.^44,45^ While the monomer typically appears with the highest response in the chromatogram, the trimer is considered to be the more specific and, hence, more reliable pyrolysis product for quantification of PS.^29,30^ As the pyrolysis efficiency can differ due to matrix effects, the use of an internal standard (ISTD) with similar properties is recommended. Here, we relied on fluorinated PS.^46^ Representative chromatograms of blanks, calibration points and samples as well as mass spectra are shown in Figure S4 of the Supporting Information.

Due to the labor-intensive sample preparation for Py-GC-MS analysis, we focused on the highest particle dose where fluorescence microscopy detected the most pronounced particle uptake. A549 cells were seeded as described above and subsequently incubated for 24 h with 31 μg·cm^–2^ fluorescently labeled spherical PS particles in DMEM. After trypsinization, cells were counted manually on C-Chip Neubauer microscopy slides (Carl Roth GmbH + Co. KG, Karlsruhe) followed by centrifugation of an aliquot of 200,000 cells which were washed twice with PBS. After centrifugation (400 rcf, 10 min) of the counted cells, the pellets were resuspended in 10 μL Milli-Q water and transferred to double-open pyrolysis glass tubes connected to transport adapters (all from Gerstel) and stuffed with quartz wool, which was previously sterilized over a gas burner for about 5 s and compressed to occupy approximately one-fifth of the tube. The source tube with the resuspended cell pellet was subsequently rinsed with 10 μL of Milli-Q water, and the rinse was transferred to the quartz wool to ensure quantitative sample transfer. The samples in the pyrolysis tubes were dried for 3 h at 50 °C in an oven. Subsequently, a 10 μL solution of the internal standard (ISTD) in dichloromethane (DCM) was added to the samples, which were dried for an additional 1 h at 50 °C in an oven. Thereafter, the transport adapters were loaded to the autosampler tray for Py-GC-MS.

Pyrolysis was performed using a thermal desorption unit (TDU 2) equipped with a pyrolysis-module (both from Gerstel, Mühlheim, Germany). The TDU was connected to a Gerstel cold injection system (CIS 4) connected to a 7890 series GC coupled with a 5975 series mass selective detector (both Agilent, Waldbronn, Germany). The samples were pyrolyzed for 1 min at 640 °C under a constant helium flow of 44 mL·min^-1^. The transfer temperature from the TDU to the CIS was 320 °C. Pyrolyzed samples were cryo-focused with liquid nitrogen at −50 °C in the CIS equipped with a liner filled with deactivated glass wool (Gerstel). After pyrolsis, the CIS was heated to 320 °C at 12 °C·s^–1^ and then held at 320 °C for 10 min. A 3:1 split was appliedfor transfer onto the HP-5MS low polarity capillary GC column ((5%-phenyl)-methylpolysiloxane, Agilent 19091S-433) with a length of 30 m, an inner diameter of 0.25 mm and a film thickness of 0.25 μm. The oven temperature gradient started at 50 °C for 2 min, followed by heating to 250 °C at 5 °C·min^-1^ and to 320 °C at 40 °C·min^-1^, which was kept constant for 13 min.

The temperatures of the GC-MS interface and the ion source were both set to 230 °C. The quadrupole was operated at 150 °C. The electron ionization energy was 70.3 eV. Each mass spectrum was obtained in combined SIM/scan mode over a mass scan range of *m*/*z* 50–500. PS-specific ions of the trimer were detected in the scheduled selected ion monitoring (SIM) at *m*/*z* 91 (quantifier), 117 (qualifier, 27% of quantifier, 20% allowed variance), and 207 (qualifier, 13% of quantifier, 20% allowed variance) to obtain extracted ion chromatograms (EICs) for PS and at *m*/*z* 97 (only one ion could be used due to too low and nonspecific signals) for the internal standard 4F-polystyrene. For calibration, PS was weighed and dissolved in dichloromethane. A 7-point calibration was prepared using 4-F-polystyrene as ISTD. Data was processed using MassHunter software (versions B.06.00 and B.05.00, Agilent). Samples for recovery experiments were prepared by transferring 200,000 cells without previous incubation with PS-NPs as described above, and spiking calibration standards of different polymer masses together with ISTD into the cell-pellet loaded pyrolysis tube. Recovery was calculated by dividing the determined polymer mass by the spiked amount.

### 2.8 Determination of the Limits of Detection and Quantification and Statistical Analysis

Limits of detection (LOD) for automated fluorescence imaging microscopy and Py-GC-MS analysis of PS were calculated by measuring ten blank samples (cells not incubated with PS-NPs) and dividing the SD of the blank sample results by the slope of the calibration curve, multiplied by a factor of 3.9 as described in DIN 32645:2008-11.^47,48^ Limits of quantification (LOQ) were calculated by multiplying the LOD with a factor of 3.3.

Data processing and further statistical analysis was performed using GraphPad Prism 10 (Boston MA, USA). For automated fluorescence imaging microscopy results the statistical analysis was performed by applying one-way ANOVA as multiple comparisons following Tukey’s test (* = *p* < 0.05, ** = *p* < 0.01, *** = *p* ≤ 0.001). For comparison of automated fluorescence imaging microscopy and Py-GC-MS an unpaired parametric *t* test (95% confidence level, two-tailed, * = *p* < 0.05, ** = *p* < 0.01, *** = *p* ≤ 0.001) was applied.

## 3 Results and Discussion

### 3.1 Particle Characterization

The characterization of particles to be investigated in toxicological assays is essential for a meaningful comparison with data from other studies. As shown in Figure 1A the mean particle diameter of spherical PS-NPs according to the supplier (180 nm) was confirmed by DLS (mean hydrodynamic diameter: 183 nm, D10: 138 nm, D50: 186 nm, D90: 251 nm), which also indicates a low degree of polydispersity (PDI: 0.014). The particle diameter was further ascertained by SEM (D10: 160 nm, D50: 176 nm, D90: 198 nm, Figure 1B) which also revealed a spherical particle morphology (Figure 1C). The zeta potential of the particles in water is negative (−44 ± 1 mV, Figure 1D), consistent with the supplier information that the nanoplastics are unfunctionalized polystyrene particles dispersed in water with a low concentration of sodium azide and no intentionally added surfactant. The dispersion thus exhibits a moderately to strongly negative zeta potential of approximately −40 mV at neutral pH, in line with literature reports for “plain” polystyrene latexes where effective negative surface charge typically arises from ionizable groups associated with the emulsion polymerization process (e.g., persulfate initiator fragments and ionizable chain ends) and residual surface-active species, rather than from deliberately introduced functional groups.^49^ When the particles are transferred into complete cell culture medium, the zeta potential shifts to −11 ± 2 mV, which is consistent with partial screening of surface charges and the formation of a protein-rich corona that masks the particle surface. Despite this change in apparent surface charge, DLS measurements in medium over 24 h did not reveal any pronounced increase in hydrodynamic diameter (Figure 1E), indicating that the particles remain colloidally stable under exposure-relevant conditions, presumably because steric stabilization by the adsorbed biomolecular corona counteracts agglomeration even at reduced electrostatic repulsion.

**Figure 1:**
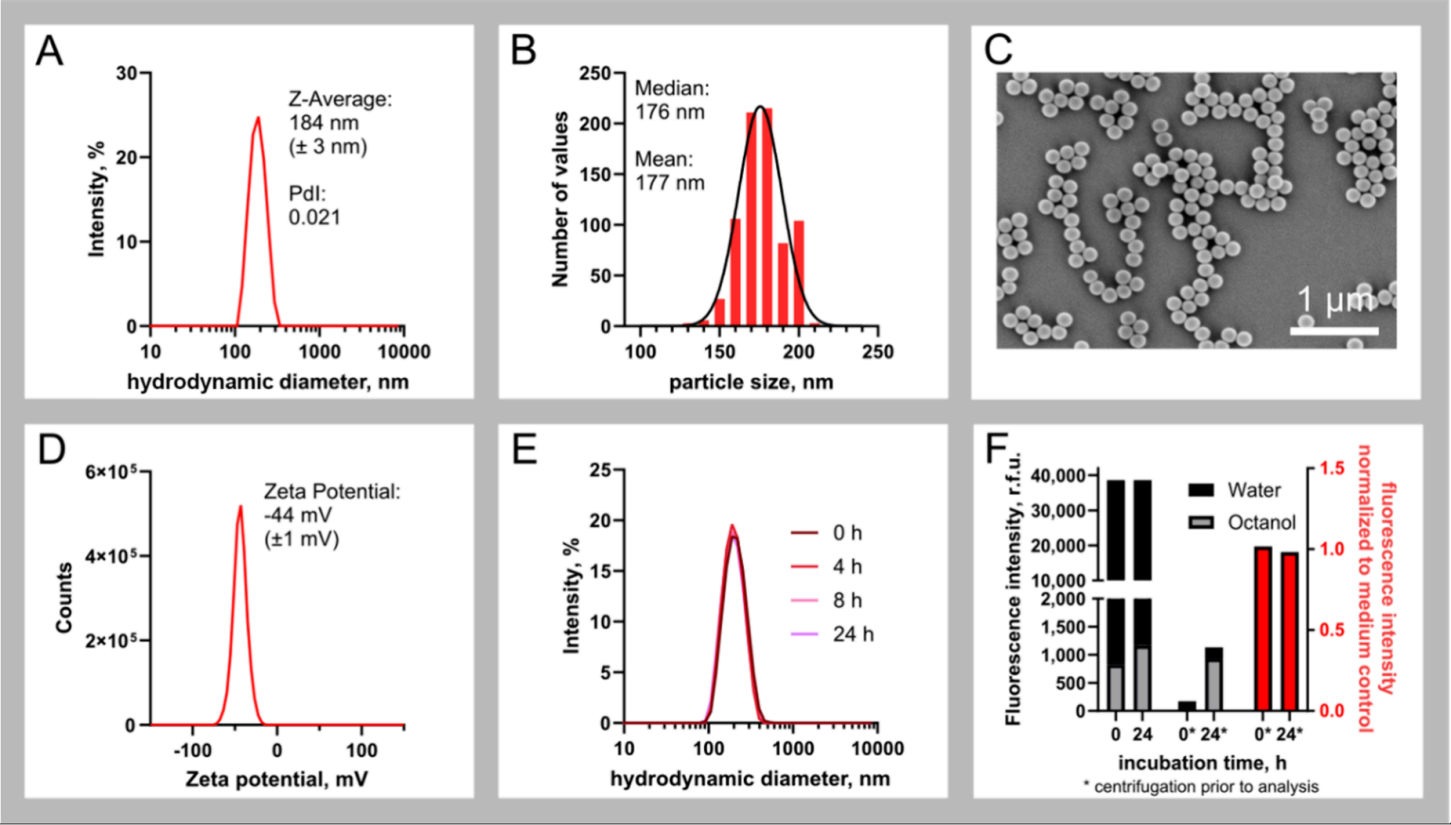
Characterization of PS nanoplastic particles. (A) Representative results from triplicate DLS measurements of a 100 μg·mL^–1^ dispersion of fluorescently labeled PS-NPs in Milli-Q water including the calculated hydrodynamic diameter (Z-average) and polydispersity index (PDI). (B) Histogram of the particle size distribution derived from SEM images. (C) Representative SEM image. (D) Representative results from triplicate zeta potential measurements (100 μg·mL^–1^ dispersion in Milli-Q water). (E) Average results from DLS measurements of at least two samples of a 100 μg·mL^–1^ dispersion in cell culture medium after incubation at 37 °C for 0, 4, 8, and 24 h. (F) Left *y*-axis: Distribution of fluorescence intensities (*λ_ex._* = 485 nm, *λ_em._* = 530 nm) in water and octanol after 0 and 24 h of incubation of PS-NPs before and after (*) centrifugation of both phases to separate PS-NPs from leached fluorophore. Right *y*-axis: Fluorescence intensity of particle-free supernatants after incubation of PS-NPs in complete cell culture medium for 0 and 24 h, normalized to the respective medium control.

To assess possible leaching of the incorporated fluorophore (Figure 1F), PS-NPs were shaken for 24 h in a biphasic system comprised of water and *n*-octanol. When analyzing the fluorescence of both phases without prior centrifugation, a clear accumulation of fluorescence in water was observed, reflecting favorable partitioning of PS-NPs to the aqueous phase likely due to similar densities (*ρ*_PS_ = 1.09 g·cm^–3^).^41^ When both phases were centrifuged to remove the particles prior to fluorescence analysis, only minor increases in fluorescence intensities were observed after 24 h. This indicates minimal leaching of the fluorophore into water. We additionally assessed fluorophore leaching in complete cell culture medium under exposure-relevant conditions. After 24 h incubation and subsequent centrifugation of the nanoparticle suspension, no increase in fluorescence was detected in the particle-free supernatant compared to the initial signal, indicating that dye release in this medium remained negligible over the incubation period.

### 3.2 Particle Dose Regime Evaluation by Cell Viability Assessment and Qualitative Characterization of Particle Uptake

Cell viability testing of different particle doses before characterizing and quantifying particle uptake *in vitro* is necessary to ensure that the applied doses do not induce cytotoxic effects that could confound the interpretation of the quantification results.^50^

Cell viability was determined with the MTT assay after 24 h incubation with 0.31–31 μg·cm^–2^ fluorescently labeled PS-NPs corresponding to concentrations of 1–100 μg·mL^–1^, which is within the range recommended by the OECD in the context of *in vitro* toxicity testing of manufactured nanomaterials.^51^ No effects on cell viability were observed up to the highest dose tested (Figure 2A). Hence, unless stated otherwise, 31 μg·cm^–2^ (100 μg·mL^–1^) was selected as the standard dosing concentration for all experiments as the resulting uptake reliably falls within the detection and quantification limits of the applied analytical methods. Moreover, the absence of any detectable decrease in cell viability in the MTT assay indicates that the incorporated fluorophore does not exert relevant toxicity under the conditions tested.

**Figure 2:**
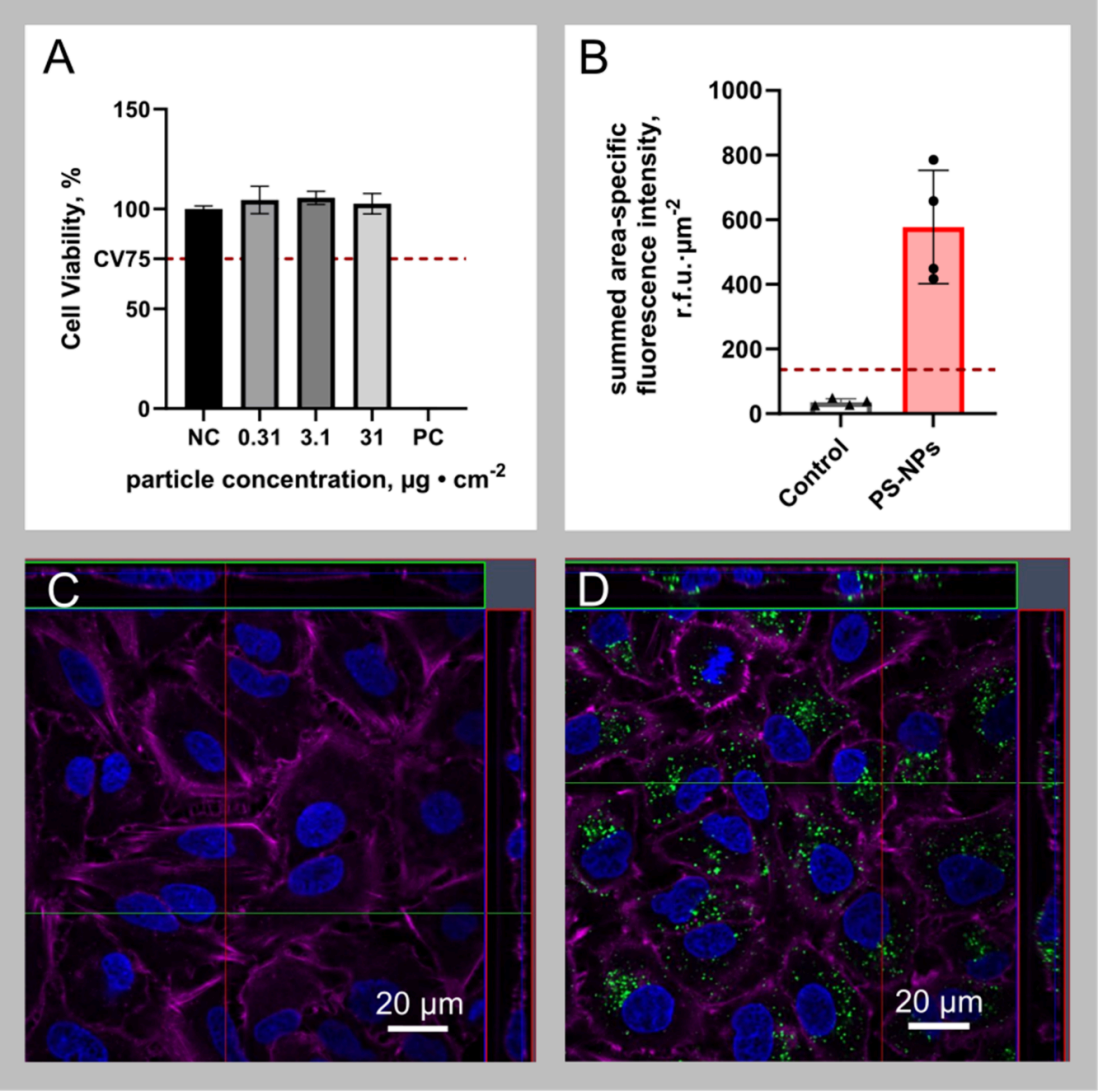
Results of MTT assays and characterization of the uptake of fluorescently labeled PS-NPs in A549 lung cells with confocal fluorescence microscopy. (A) Cell viability after incubation of A549 lung cells for 24 h with increasing concentrations of spherical fluorescently labeled PS-NPs. Medium containing 10% Milli-Q water and 0.1% Triton-X-100 were used as negative (NC) and positive controls (PC), respectively. The red dotted line indicates the critical value of a cell viability of 75%. Error bars indicate the SD of three independent biological replicates. (B) Mean of the summed area-specific fluorescence intensities of the fluorophore incorporated in PS-NPs per z-stack (approximately 20–30 cells per stack, averaged over 2–4 z-stacks) for untreated (control) and treated cells. The red dashed line indicates the uptake threshold of 136.6 r.f.u.·μm^–2^. Dots represent independent biological replicates, which were each averaged over two technical replicates. Error bars indicate the SD of four biological replicates. Representative z-stack images of (C) untreated cells (control) and (D) cells incubated with 31 μg·cm^–2^ PS-NPs for 24 h. Magenta represents cellular actin structures (stained with Alexa Fluor 647 Phalloidin), blue represents the nuclei (stained with Hoechst 33342), and green represents the fluorophore incorporated in the PS-particles. The green and red lines in panels (C, D) indicate horizontal and vertical cuts through the image, respectively, with the corresponding horizontal and vertical views shown in the green and red boxes around the main images.

In contrast to conventional fluorescence microscopy, confocal microscopy allows for imaging of samples in three dimensions. Computational processing of measured height-dependent fluorescence intensities produces 3D images (z-stacks). To determine an intensity threshold that confirms uptake of fluorescent PS-NPs in treated A549 cells, ten samples of untreated cells (blanks) were analyzed by confocal microscopy. After automated image processing (described in detail in Section 2.4) the mean and SD of the sum of the fluorescence intensities for each z-stack recorded in the emission channel specific for the particle-incorporated fluorophore per cell-associated area within the actin structure stained with Alexa Fluor 647 Phalloidin were calculated. The threshold was defined as the mean of all ten blank sample results plus ten times the SD,^52^ resulting in a value of 137 r.f.u.·μm^–2^ (dashed line in Figure 2B). Although this is a rather conservative approach compared to typical cut-offs of 2–3 SDs, we deliberately chose this stringent criterion to exclude cells with high background fluorescence, ensuring that only unequivocally positive signals are classified as true uptake events.

A549 cells that were incubated with 31 μg·cm^–2^ fluorescently labeled PS-NPs for 24 h showed a mean area-specific fluorescence intensity of 600 ± 200 r.f.u.·μm^–2^ (Figure 2B), which is 4.2-fold higher than the threshold value and 17.3-fold higher than the untreated control. Significant uptake could also be visibly confirmed in the z-stack images (compare Figure 2C and Figure 2D). Notably, this method does not allow to clearly distinguish between particles located inside or directly outside of membranous structures. Therefore, in this context, “uptake” refers to both, intracellular particles and particles involved in cellular interactions, which might be internalized at later stages. The risk of including extracellular particles in the analysis was minimized by (i) proper washing of the cells prior to analysis (3 × PBS) and (ii) including only areas that show colocalization with the Actin stain, indicating that these particles are embedded in intracellular structures.

In order to determine the proportion of cells which took up nanoparticles, the samples were analyzed via flow cytometry. Flow cytometry allows for automated analysis of fluorescence at different emission wavelengths in individual cells and has been used to semiquantitatively characterize uptake of labeled MNPs *in vitro*.^21^ In this work, the technique is applied to determine the proportion of A549 cells exhibiting increased fluorescence due to particle uptake. We included immunostaining of cells prior to analysis to exclude contributions to the PS-NP-associated fluorescence signal in the FITC channel from noncell-based structures (see Section 2.5).^40^ The results show a clear shift in mean fluorescence intensity from 900 ± 500 r.f.u. for untreated A549 cells to 10,200 ± 1,400 r.f.u. for cells treated with 31 μg·cm^–2^ fluorophore-labeled PS-NP for 24 h (Figure 3A). After subtracting the percentage of events corresponding to the far end of the blank samples above the threshold, it was confirmed that 98.1% ± 0.3% of the antibody-stained single cells can be considered positive in terms of MNP uptake (Figure 3B). The possibility that cells with particles that are associated with the outer cell membrane that are not (yet) internalized are included in those events needs to be considered. However, Salvati et al. determined only a minor contribution due to surface-adhered fluorescent PS particles on A549 cells in flow cytometry analysis if a sufficient number of washing steps (here: 3 × PBS) was included.^22^

**Figure 3:**
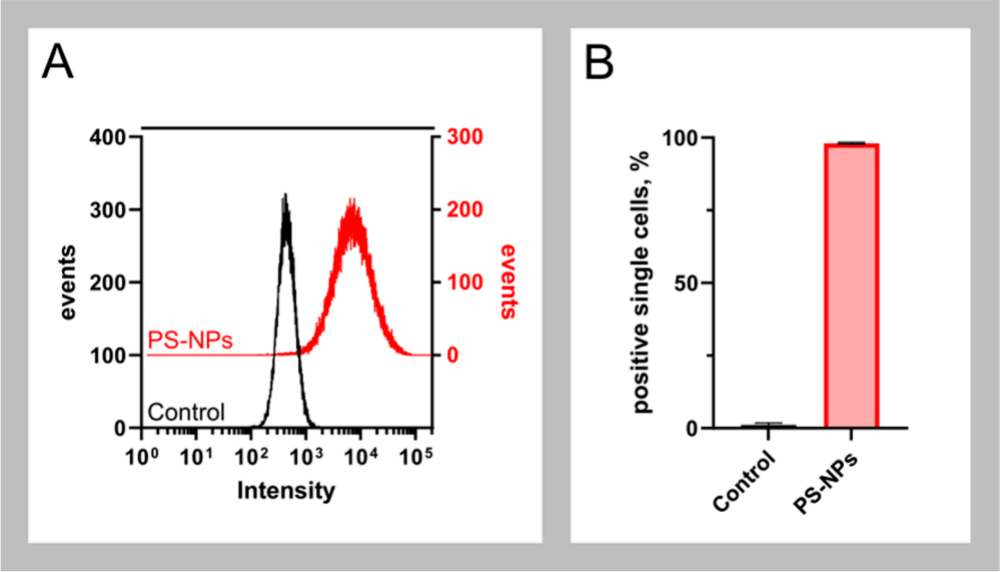
Flow cytometry results for A549 lung cells exposed to fluorescently labeled PS-NP at 31 μg·cm^–2^ for 24 h indicating particle uptake by almost all cells. (A) Representative histograms representing the FITC channel (representing the fluorophore incorporated in PS-NPs) and (B) corresponding percentage of positive single cells above the threshold set at an intensity of 10^3^ r.f.u. Error bars represent the SD of all nine replicates (three technical replicates within three independent biological replicates).

Additionally, the distribution of cells with respect to fluorescence intensity does not reveal any distinct subpopulations mainly driving MNP uptake. This confirms the visual evaluation of the images obtained with confocal fluorescence microscopy which indicate an uptake of particles by almost all cells, with some taking up more particles than others. Furthermore, when assessing the side scatter plot for events with low and high emissions, a clear shift in granularity becomes apparent (see Figure S1B,C). Increased side scattering was previously reported as a marker for cellular uptake of nanomaterials, providing options to assess unlabeled materials by flow cytometry *in vitro*.^53,54^

### 3.3 Quantitative Analysis of Particle Uptake via Automated Fluorescence Imaging Microscopy and Pyrolysis Gas Chromatography Mass Spectrometry (Py-GC-MS)

Fluorescence microscopy has frequently been used for characterizing cellular uptake of fluorescent particles *in vitro*,^19,55–58^ yet no reliable approach for absolute quantification has been reported. Here we developed an automated read-out which, in combination with an external calibration, facilitates high-throughput testing with decreased operator bias and therefore higher expected reproducibility compared to conventional fluorescence microscopy.^59^

The scheme in Figure 4 depicts the workflow to determine the average particle uptake per cell. In this approach, A549 cells were incubated with doses of 3.9–31 μg·cm^–2^ fluorescently labeled PS-NPs for 24 h in a 96 well plate, and then washed and stained for actin and nuclei. The cells were subsequently analyzed in a high-content fluorescence imaging microscope, where five images per well were automatically selected by the imaging software for further processing (see Section 2.6 for details; representative images are provided in Figure S5). Outlining all cellular regions (actin) and nuclei (Hoechst) within each image (Figure 4A) allows for the calculation of parameters including the total cell-associated area, the number of nuclei—and hence, of cells—and the fluorescence intensity of the fluorophore associated with the particles that are located within the outlined cell area. An external calibration with labeled PS-NPs (Figure 4B) translates measured fluorescence intensities into the average cell-associated polymer content, which can be converted into the average particle number per cell. Therefore, we approximated the particle volume and corresponding particle mass of 3.33 fg from the mean diameter (see Section S3), assuming spherical particles with homogeneous density. This can be justified given the low PDI of the investigated PS-NPs, however, the contribution of rare larger particles might lead to an overestimation of the particle number. Additionally, MNPs with pronounced polydispersity or locally varying effective densities, e.g., due to weathering, would require a more detailed size-resolved analysis.

**Figure 4:**
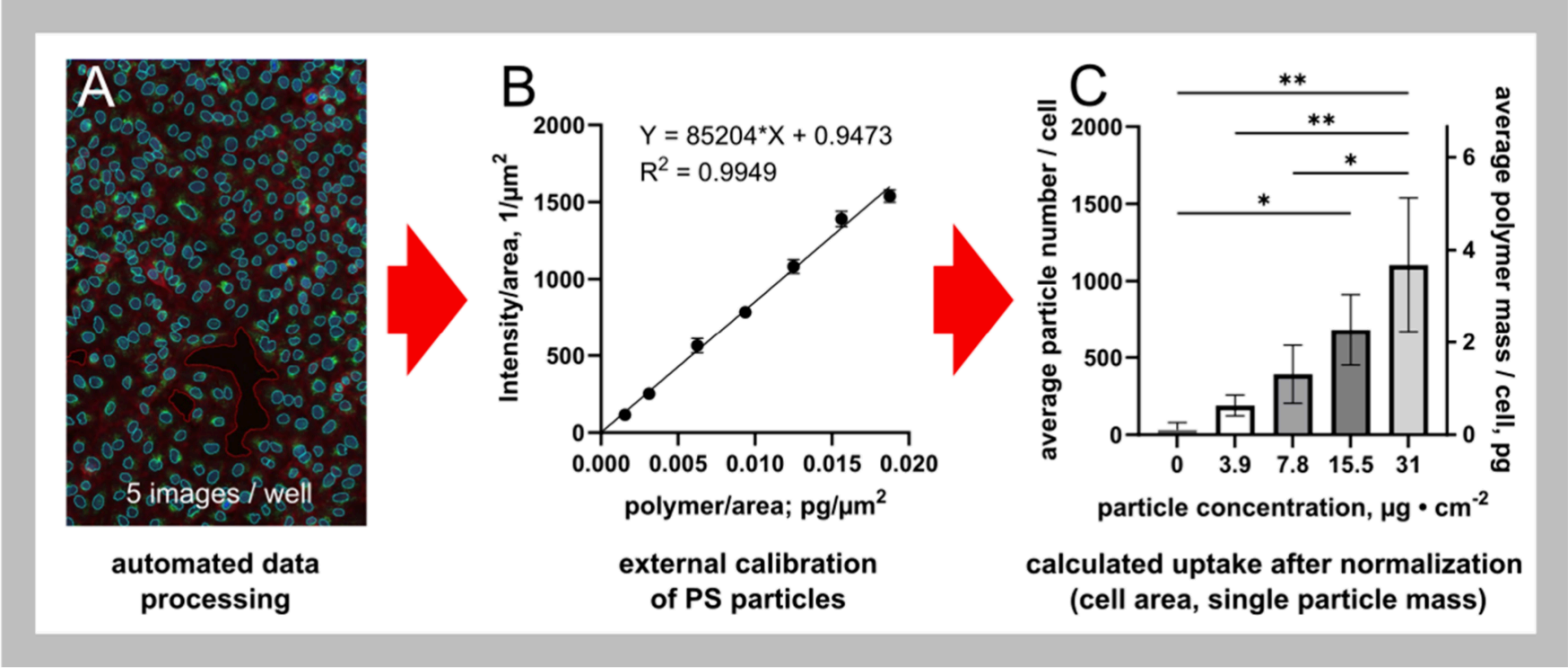
Quantifying uptake of fluorescently labeled PS-NPs in A549 lung cells with automated fluorescence imaging microscopy. (A) Representative image during data processing. Regions representing cellular actin structures (stained with Alexa Fluor 647 Phalloidin) and nuclei (stained with Hoechst 33342) are outlined in red and blue, respectively. The fluorophore incorporated in the PS-particles is highlighted in green. (B) Representative external calibration of PS-NPs for quantifying cellular uptake, featuring area-specific fluorescence intensity as a function of polymer mass. (C) Average particle numbers and polymer masses taken up per A549 cell after incubation with increasing concentrations of PS-NPs for 24 h. Error bars indicate the SD of three independent biological replicates (* = *p* < 0.05, ** = *p* < 0.01).

A dose-dependent uptake of fluorescent PS-NPs in A549 cells was quantified by the devised automated fluorescence imaging microscopy method. The lowest particle dose of 3.9 μg·cm^–2^ resulted in an average uptake of 190 ± 70 particles per cell, which is clearly above the LOQ of 74 particles per cell (LOD: 22 particles per cell, also see Section 2.8 and Table 1). Higher doses of 7.8 and 15.5 μg·cm^–2^ yielded average particle numbers of 400 ± 200 and 700 ± 250 per cell, respectively. Finally, when incubated with the highest tested dose of 31 μg·cm^–2^, each cell harbored an average of approximately 1100 ± 450 particles after 24 h (Figure 4C). Doubling the area-specific dose from 15.5 to 31 μg·cm^–2^ increased the cellular uptake by only about 60%, indicating a deviation from linearity at higher doses. This might suggest a saturation effect under the applied experimental conditions, although the comparatively large standard deviations warrant to interpret this trend with caution. A nonlinear relationship between externally applied and internalized dose would imply that dosimetric assessments based solely on nominal exposure may be misleading and that direct quantification of the dose that actually has been taken up is essential for a meaningful interpretation of dose–response relationships. Potential contributing factors to uptake saturation include finite numbers of cellular binding and internalization sites, particle sedimentation resulting in altered effective particle concentrations at the cell surface at higher applied doses, and transport limitations that restrict further cellular uptake once a substantial fraction of the deposited particles has already interacted with the cell layer.

**Table 1:** Comparison of LOD, LOQ, and Results for the Quantification of Particle Uptake in A549 Lung Cells Obtained with Automated Fluorescence Imaging Microscopy and Py-GC-MS^a^

method	LOD (particles per cell)	LOQ (particles per cell)	quantification results (particles per cell)	corrected results (particles per cell)
automated fluorescence imaging microscopy	22	74	1100 ± 450	1300 ± 500
Py-GC-MS	8	26	950 ± 200	1700 ± 350

a Corrected results take into account an underestimation of 20% for automated fluorescence imaging microscopy and a recovery rate of 57% for Py-GC-MS. Cells were incubated with PS-NPs at 31 μg·cm^–2^ for 24 h.

Experiments employing formaldehyde fixation of the cells prior to incubation with PS-NPs in order to exclude active uptake indicate that membrane-associated particles make up only 2–12% (from the lowest to the highest applied dose) of the particle count determined by automated fluorescence imaging microscopy (Figure S6). Even if this system cannot fully mimic the membrane-particle interactions encountered with living cells, the results suggest only a minor contribution of membrane-adhered PS-NPs to the total uptake. A complete separation of internalized and purely surface-adherent particles remains experimentally challenging, particularly for nanoscale materials and over longer exposure times. Consequently, the uptake values reported here should be interpreted as measures of the total particle content associated with cells.

The workflow for the detection and quantification of PS in A549 cells with Py-GC-MS (see Section 2.7) resulted in LODs and LOQs of 5.2 and 17.0 ng PS. This corresponds to an average of 8 and 26 particles per cell, respectively, and is slightly lower than the values obtained from automated fluorescence imaging microscopy (Table 1). Applying external calibration for quantification (Figure 5A) the average polymer uptake in 200,000 A549 cells incubated with 31 μg·cm^–2^ PS-NPs for 24 h was 650 ± 100 ng PS, which corresponds to 950 ± 200 particles per cell (Figure 5B). This is in good agreement with the results obtained by the automated fluorescence imaging microscopy method described above (1100 ± 450 particles per cell, < 15% deviation). The reported uncertainties of the results from each method are derived from the analysis of different biological replicates (different passage numbers) on different days, thus providing a realistic estimate of the intermediate precision of the two methods.

**Figure 5:**
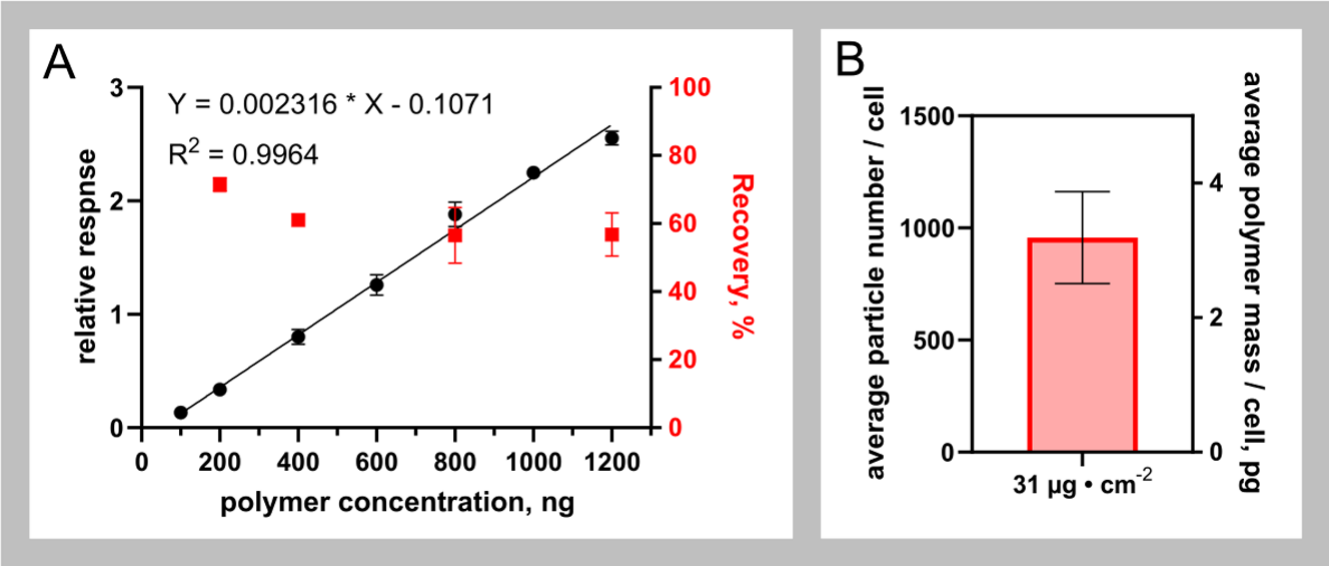
Quantification of PS-NP uptake in A549 lung cells via Py-GC-MS. (A) Representative external calibration of polystyrene (PS) using 4F-polystyrene as internal standard. Recoveries obtained from blank matrix samples spiked directly before Py-GC-MS with different amounts of PS standard are shown in red. (B) Average particle and polymer uptake per cell after incubation with 31 μg·cm^–2^ PS-NP for 24 h. Error bars indicate the SD of three independent biological replicates.

Quantification of nanoplastic uptake *in vitro* involves several analytical challenges that may contribute to both underestimation and overestimation of true uptake levels. To increase specificity and reduce false positives, we specifically chose the styrene trimer for quantification, as it is a more specific marker compared to the often-referenced monomer.^34^ Although Py-GC-MS allows for direct analysis of cells and therefore minimizes losses due to sample handling, matrix effects from complex biological material can interfere with polymer detection, leading to reduced sensitivity and recovery rates despite method optimization.^33^ Recovery experiments with nontreated but processed cells that were spiked with dissolved PS directly before Py-GC-MS indicate an underestimation of the quantified amount of PS. Specifically, recoveries across the tested concentration range were between 57 and 71% (see Figure 5A), with both 800 ng and 1200 ng PS spikes yielding recoveries of 57%. The cell-associated polystyrene signal in the uptake experiments fell between these two calibration points, consistent with a linear response in this concentration regime. For conservative quantification, absolute uptake values were therefore corrected using a recovery factor of 57%, resulting in an uptake of 1700 ± 350 particles per cell (see corrected results in Table 1). The observed incomplete recoveries most likely reflect matrix-related effects, including incomplete pyrolysis of PS NPs in the complex cellular matrix, adsorption of pyrolysis products to quartz wool or reactor surfaces, and potential losses during sample drying, particularly since the dissolved PS standard used for spiking does not perfectly mimic the behavior of solid 180 nm PS-NPs embedded in a cell pellet.

Trypsinization of cells during sample preparation for Py-GC-MS could potentially release particles that remained attached to the well surface despite extensive washing with PBS. Compared to quantification by automated fluorescence imaging microscopy, which excludes areas not associated with cellular structures, this would lead to an overestimation of particle uptake by Py-GC-MS. However, only very low residual fluorescence remained after washing well plates incubated for 24 h with the highest dose of PS-NP, which was not affected by trypsinization (Figure S7). This demonstrates that triplicate washing with PBS is efficient in removing unbound particles as previously described.^22^

On the other hand, particles adhering to the cell membrane could lead to an underestimation of the particle number determined by automated fluorescence imaging microscopy. The focal plane of the laser beam during read out does not cover the entire cell volume (autofocus uses the nuclei stain as reference), while in Py-GC-MS every particle—membrane-adhered or internalized—is included in the analysis. Considering a cell layer thickness of approximately 3–5 μm^60^ (with approximately 10–15% of cells showing a thickness of up to 9 μm, as determined by confocal microscopy) and a depth of focus (DOF) of around 4.5 μm (resulting from an objective with 20-fold magnification, 0.7 numerical aperture and 0.5-fold magnification changer),^61,62^ an estimated particle fraction of 10–15% outside the given DOF could escape from detection by automated fluorescence imaging microscopy. In future studies, the use of different objectives and apertures could increase the DOF to capture the entire cell volume. Additionally, the automated processing of the fluorescence images includes nuclei that are on the borders of the image (approximately 4–6%, counted manually). This could account for an overestimation of the cell number and therefore result in lower particle numbers per cell. When considering an aggregated underestimation of 20% (sum of the described uncertainties), the average particle uptake per cell would increase to approximately 1400 PS-NPs (Table 1). With these corrected results, the difference in particle numbers determined with both methods changes from −14.4% to +26.5%, which is still comparable within the respective uncertainties.

The PS nanoparticles used in this study are manufactured with the fluorophore incorporated throughout the polymer matrix rather than attached to the particle surface. This bulk incorporation mode is expected to make the particle-associated fluorescence less sensitive to changes in the immediate environment, because the dye molecules remain embedded in the hydrophobic polystyrene phase both on the calibration substrate (well plate) and after cellular uptake, thereby reducing direct exposure to aqueous and acidic compartments, such as lysosomes. In line with this expectation, the fluorescence of the particle suspensions did not reveal any detectable change in fluorescence intensity over a pH range from 7.4 down to 4 (Figure S8), indicating that the emission of the incorporated dye is essentially pH-insensitive under the conditions tested. Moreover, the sample preparation protocol for imaging involves fixation followed by permeabilization, which is expected to equilibrate the pH in lysosomal compartments with the surrounding medium. As a result, any residual pH sensitivity of the fluorophore would play only a minor role for the quantitative fluorescence readout used in this study.

Nevertheless, the intracellular environment can still influence the effective fluorescence emission per particle, for example through changes in the local refractive index, microviscosity, or partial degradation of the polymer matrix over prolonged incubation. Such effects would manifest as a systematic deviation between the calibration performed on particles sedimented on a plastic substrate and the true brightness of particles residing in endolysosomal compartments, and could in principle lead to an under- or overestimation of absolute uptake numbers. In the present work, the good agreement between fluorescence-based uptake estimates and the independent polymer mass quantification by Py-GC-MS suggests that these effects are limited under the chosen conditions. However, this assumption should be revisited when applying the workflow to other particle types, fluorophores, or exposure scenarios.

Considering an average volume of the cytoplasm of a single A549 cell of 1200 μm^3^ ^63^ and the volume of a 180 nm spherical PS bead of 0.003 μm^3^, 1700 PS-NPs per cell would make up roughly 0.4% (*V/V*) of the cytoplasm. Taking into account that PS particles were observed to be predominantly located in lysosomes of A549 cells after uptake,^64,65^ this would be less than the ∼1.4% (*V/V*) of the cytoplasm that lysosomes typically account for.^63^ The obtained results can therefore be considered as morphologically realistic. Finally, it should be mentioned that an exposure concentration of 100 μg·mL^–1^ is beyond those typically expected in real-life exposure scenarios. While such concentrations are appropriate to probe potential hazards and to uncover cellular responses, they likely exceed predicted environmental nanoplastic levels, which are generally estimated to be in the low μg·mL^–1^ range or below.^66,67^ Nevertheless, the applied concentrations are justified as they enable robust quantification of cellular uptake, providing an essential worst-case benchmark for hazard assessments and may guide future studies at lower, more environmentally realistic doses.

The use of A549 lung cells, a transformed alveolar epithelial cell line that does not fully recapitulate the structural and functional complexity of the human airway barrier, is a limitation of this study which aimed for comparability to and validation of previous work. Future work should therefore extend the presented workflow to primary human airway epithelial cells and to advanced cell culture systems that form more realistic barrier structures, including air–liquid interface (co)­cultures or organ-on-chip models, to better reflect *in vivo* exposure scenarios.

## 4 Conclusions

We provide a workflow that connects state-of-the-art analytical techniques for the qualitative characterization and quantification of the uptake of PS-NPs in A549 lung cells, including both the association with the outer cell membrane and actual internalization. The workflow allows for the verification of results through orthogonal methods based on fluorescence imaging microscopy and Py-GC-MS.

Cell viability testing by MTT assay showed no cytotoxic effects up to the highest administered dose of 31 μg·cm^–2^ (100 μg·mL^–1^). Confocal fluorescence imaging microscopy and flow cytometry indicated that almost all of the cells (98.1% ± 0.3%) take up fluorescently labeled PS-NPs after exposure to this concentration for 24 h. Quantification via automated fluorescence imaging microscopy revealed a dose-dependent uptake, accumulating on average 1100 ± 450 particles in each cell at the highest tested particle load. Py-GC-MS provided similar results (950 ± 200 particles per cell)—demonstrating its successful and novel application for quantifying the *in vitro* uptake of nanoplastics in human cells.

In future applications, both quantification methods developed here could allow for correlating biological effects elicited by MNPs *in vitro* with the actual amount of cell-associated particles in addition to the administered particle dose. For example, it was previously demonstrated that microplastics may release sorbed environmental contaminants and polymer additives into biological media.^68,69^ Whereas particles in the micrometer range are typically too large to be effectively taken up by cells, nanoplastics may indeed function as vectors for transporting sorbed chemicals into compartments of epithelial cells^70^ where intracellular desorption may contribute to the exposure to these substances.

Future work could build on this cell-population-level quantification method by integrating the workflow with high-resolution correlative microscopy approaches that provide subcellular information on nanoplastic localization and nano–bio interface morphology. Correlative strategies that combine advanced fluorescence imaging modalities with electron or ion microscopy and elemental mapping have recently been demonstrated for nanoparticle interactions with lung epithelial cells and would offer a powerful complement to the bulk uptake and averaged single-cell readouts established here.^71^ Such combined approaches could connect absolute particle numbers per cell to specific subcellular compartments and morphological changes, thereby further strengthening the mechanistic interpretation of dose–response relationships.

Since fluorescence-based methods are a central part of this workflow, it is so far only applicable to labeled particles. With a careful choice of fluorophores, however, mixtures of different polymer particles should be accessible. In this context, strategies involving particle staining with fluorescent protein coronas are encouraging^72^ and are currently being investigated in our laboratory. Expanding the workflow to different nanoparticle size ranges will be important to determine size-dependent uptake thresholds, while method validation across different cell types will help assessing the broader physiological relevance of our findings. Furthermore, the suitability of Py-GC-MS for the quantification of unlabeled nanoplastics makes this technique a promising tool for the analysis of mixtures of complex composition and shapes, although unspecific fragmentation during pyrolysis remains challenging for some polymers.

## Supplementary Material



## Data Availability

Processed quantitative data sets underlying the main figures (including uptake quantification, viability data, and Py-GC–MS uptake results) are available via the eNanoMapper repository (https://enanomapper.adma.ai/). All other data supporting the findings of this study are available within the article and its Supporting Information. Due to the large size and heterogeneous formats of the raw microscopy, flow cytometry, and pyrolysis-GC-MS instrument files, these are not deposited in a public repository but can be obtained from the corresponding author upon request.

## Notes

Assessment of the pH dependency of fluorescence intensity of PS nanoparticles, effects of trypsin incubation on surface-adherent particles, comparison of PS-NP uptake in A549 cells with and without formaldehyde fixation prior to incubation, representative images for automated high-throughput fluorescence microscopy, assessment of fluorophore leaching into the cell culture medium, Py-GC-MS chromatograms and mass spectra of styrene trimer and 4F-styrene trimer, the image analysis workflow for automated fluorescence microscopy, calculation of the average polymer mass per PS-NP assuming low polydispersity, and the gating strategy for flow cytometry analysis (PDF)

## List of Abbreviations

APC: allophycocyanin
CIS: cold injection system
DMEM: Dulbecco’s modified eagle medium
DMSO: dimethyl sulfoxide
DNA: DNA
DOF: depth of focus
DSMZ: Deutsche Sammlung von Mikroorganismen und Zellkulturen
ISTD: internal standard
LOD: limit of detection
LOQ: limit of quantification
MNP: micro and nanoplastic particle
MP: microplastic particle
MTT: 3-(4,5-dimethylthiazol-2-yl)-2,5-diphenyltetrazoliumbromid
NP: nanoparticle
OECD: Organization for Economic Co-Operation and Development
Py-GC-MS: pyrolysis gas chromatography mass spectrometry
PS: polystyrene
r.f.u.: relative fluorescence units
SC-ICP-MS: single-cell inductively coupled plasma mass spectrometry
SD: standard deviation
TDU: thermal desorption unit
3D: three-dimensional
