# Supplementary material for: Quantifying Cellular Uptake of Nanoplastics In Vitro: A Workflow for Fluorescently Labeled Nanosized Polystyrene Particles
Source: ACS Omega. 2026 Jul 27;11(31):46105–18. doi: 10.1021/acsomega.6c02589 (PMC13470744; doi:10.1021/acsomega.6c02589)
Supplement: Supplementary file 1 [file ao6c02589_si_001.pdf]

Supporting Information

Quantifying cellular uptake of nanoplastics *in vitro*

A workflow for fluorescently labeled nanosized polystyrene particles

Authors: Markus J. Kirchner[1,2], Francesca Bennet[1], Adriaan J. A. Duijndam[3], Florian Meirer[3], Christian Laforsch[2], Alexander Roloff[1*]

[1] German Federal Institute for Risk Assessment (BfR), Department of Chemical and Product Safety, Max-Dohrn-Str. 8-10, 10589 Berlin, Germany

[2] University of Bayreuth, Animal Ecology I, Bayreuth Center of Ecology and Environmental Research (BayCEER), Universitätsstr. 30, 95447 Bayreuth, Germany

[3] Utrecht University, Inorganic Chemistry and Catalysis, Institute for Sustainable and Circular Chemistry, Universiteitsweg 99, 3584CG Utrecht, The Netherlands

S1 Gating strategy for flow cytometry analysis

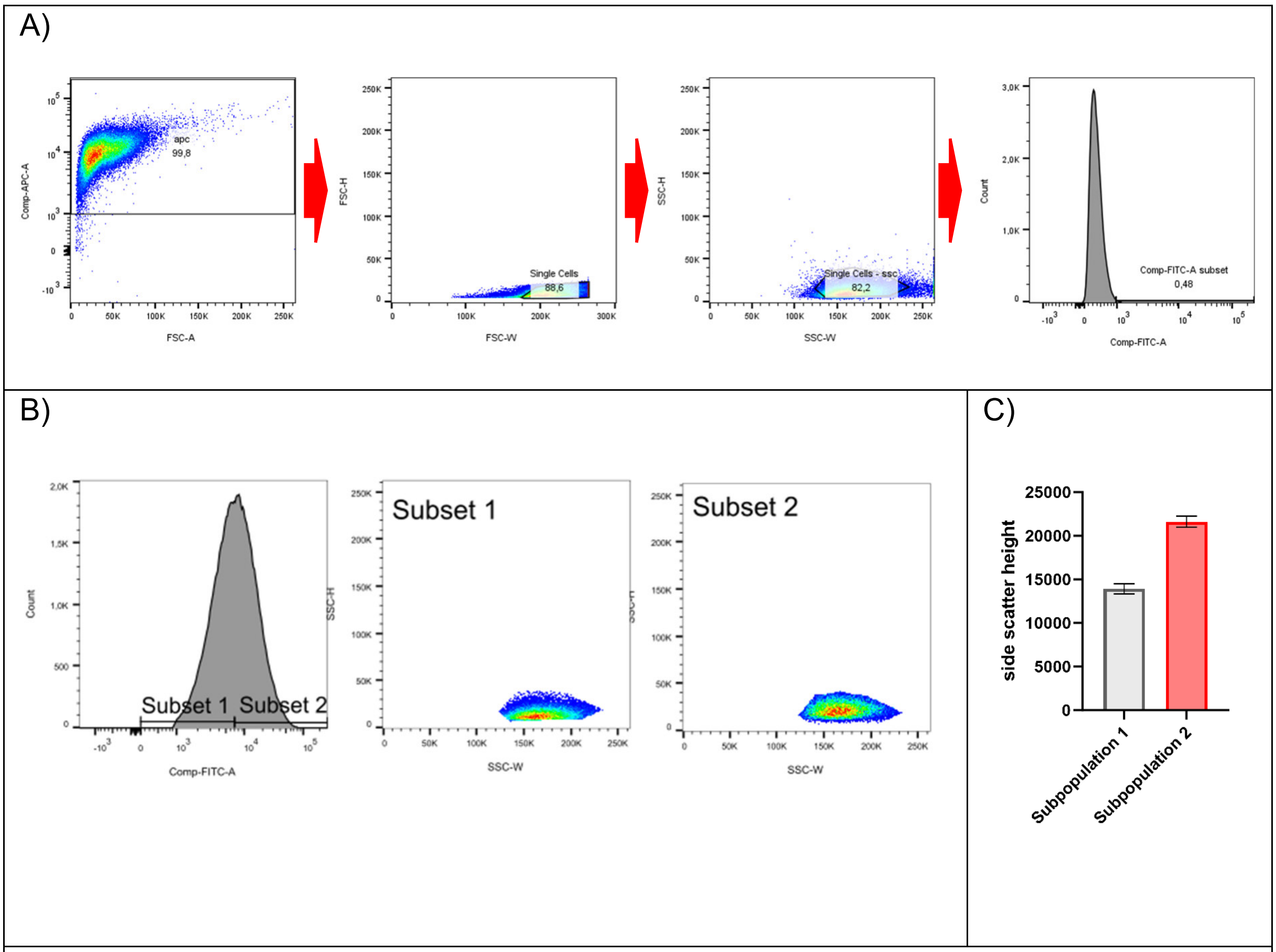

Fig. S1: Gating strategy for flow cytometry analysis. Firstly, a gate in the APC (Allophycocyanin) channel was applied to extract all CD58-positive cells. Secondly, it was gated by forward scatter and finally by sideward scatter to extract all single cells for further analysis for FITC intensity. A) Representative graphs of the gating strategy for untreated A549 cells. B) Representative side scatter plots for events with lower (subpopulation 1) and higher (subpopulation 2) fluorescence intensities than the intensity with the highest observed detection frequency in the FITC channel (representative for the fluorophore associated with the PS-NPs.) C) Mean values of the side scatter plots for subpopulations 1 and 2. Error bars indicate the standard deviation of 9 samples.

S2 Image analysis for automated fluorescence imaging microscopy

Replicate 1	Replicate 2
**A**<br>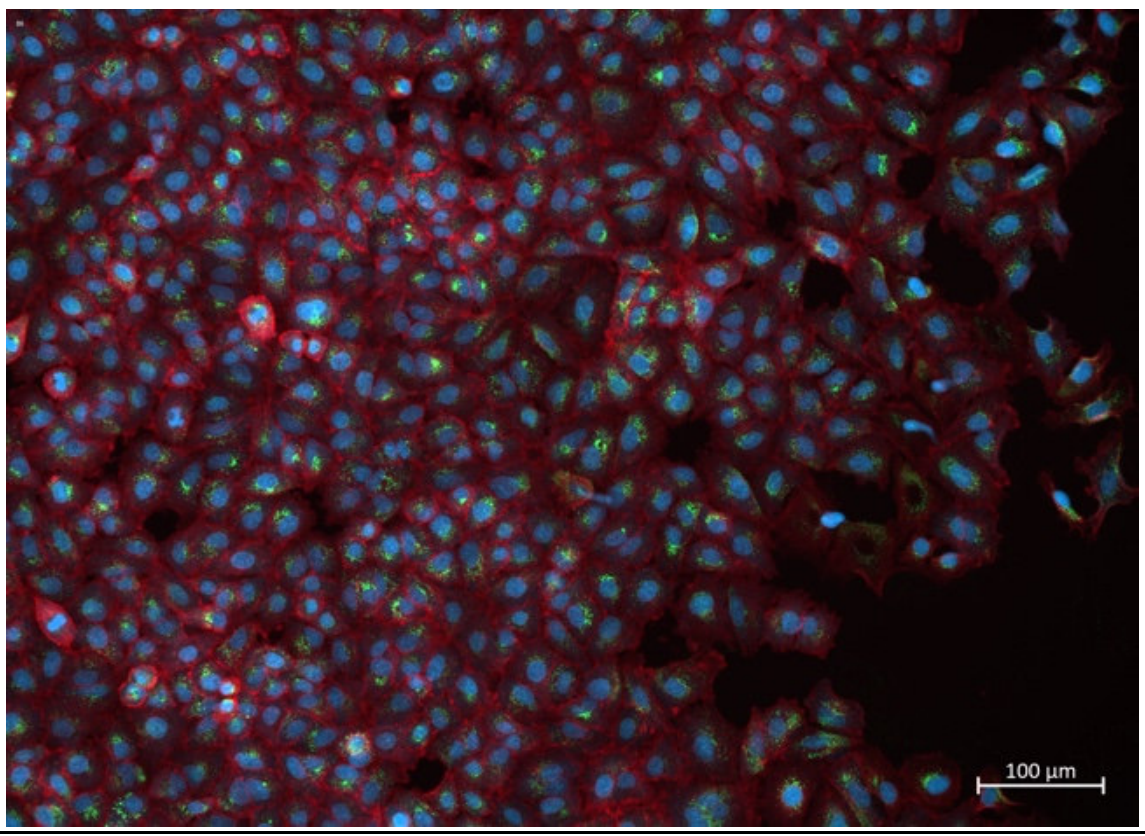	**B**<br>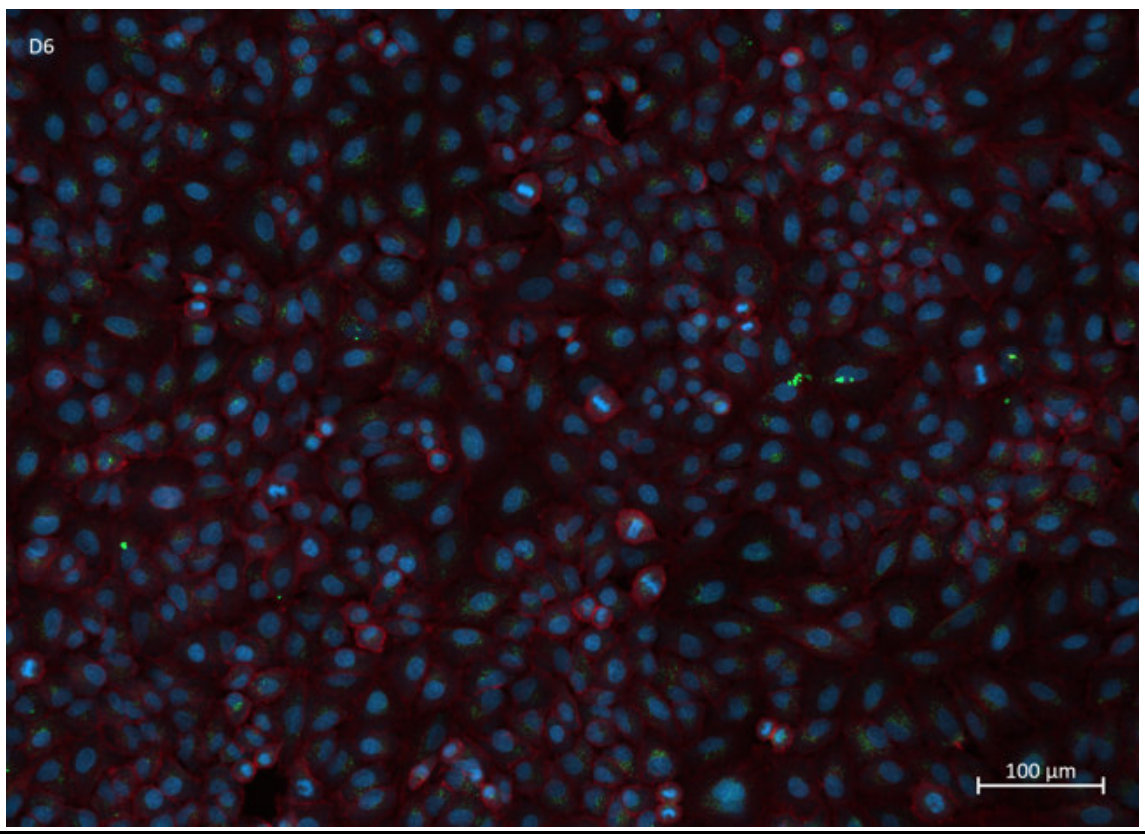
**C**<br>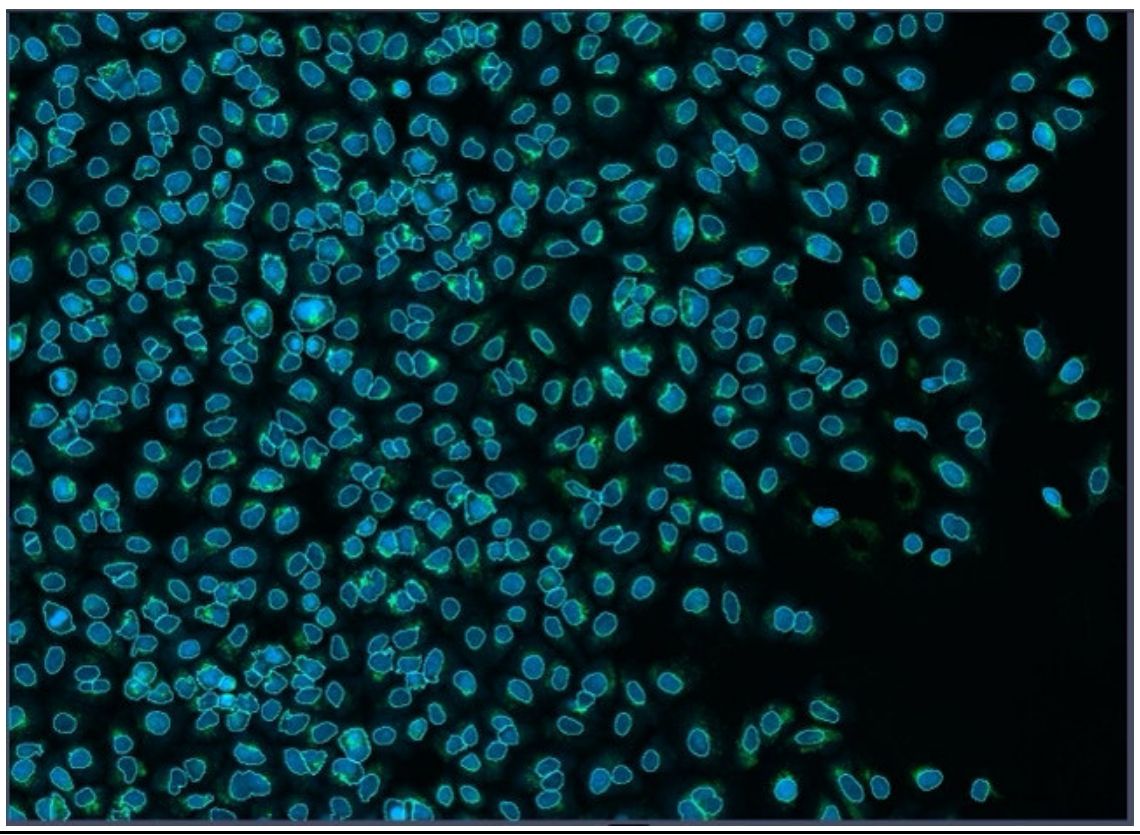	**D**<br>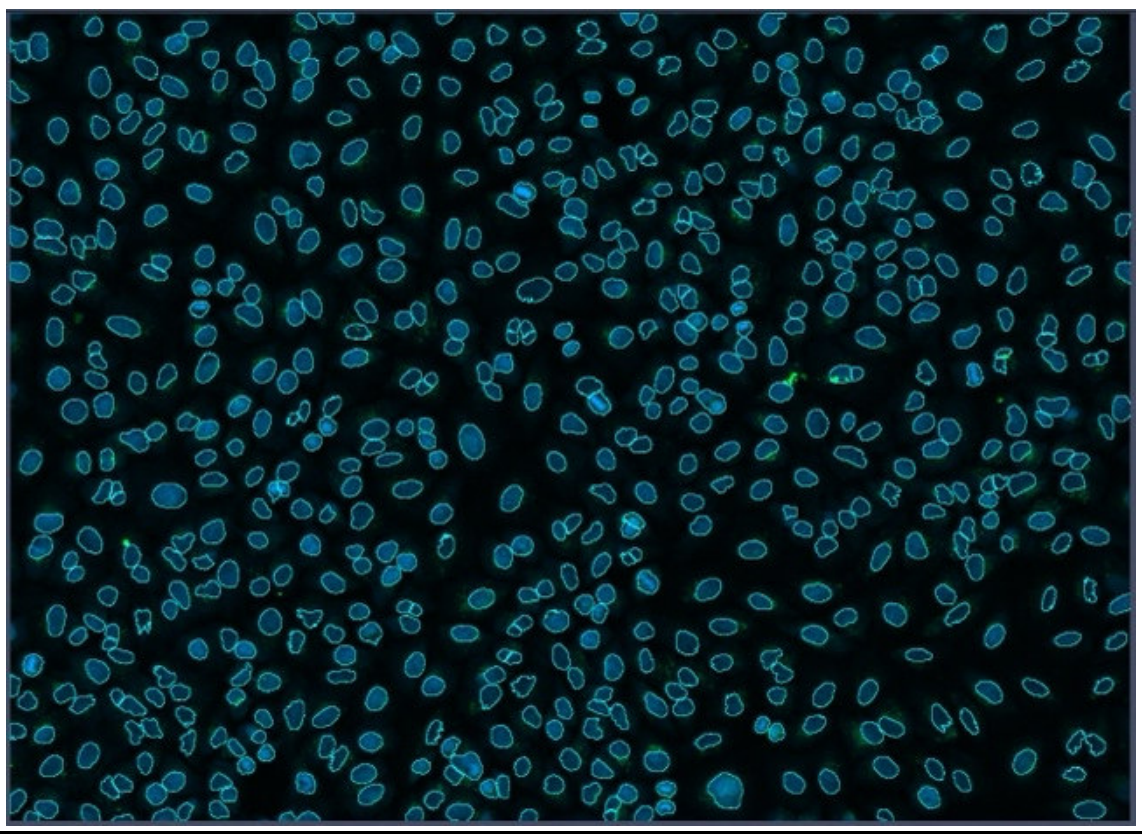
**E**<br>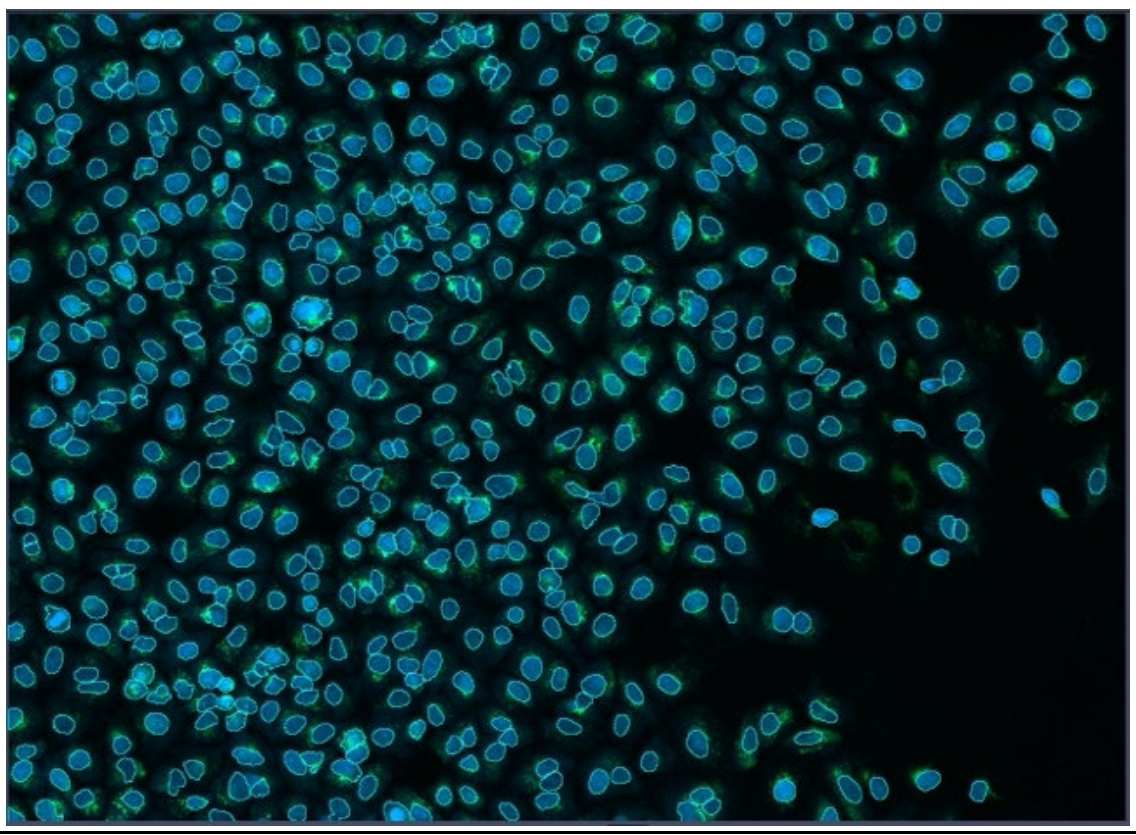	**F**<br>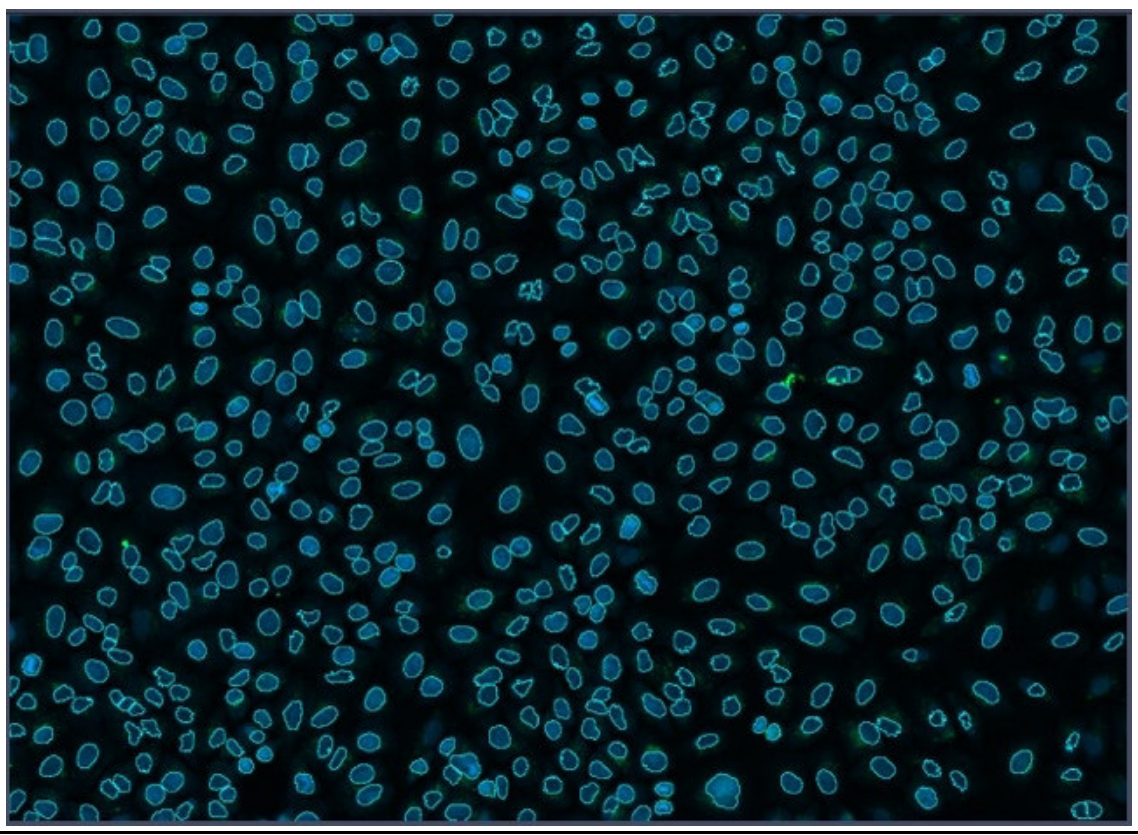
**G**	**H**

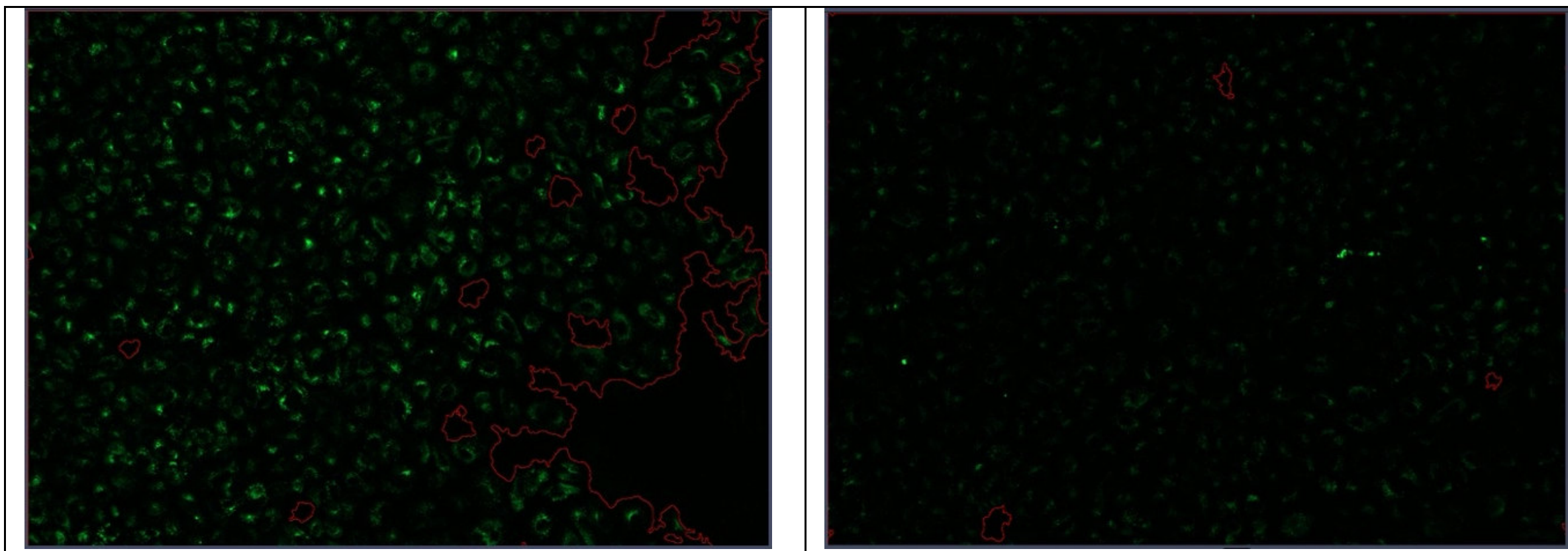

I

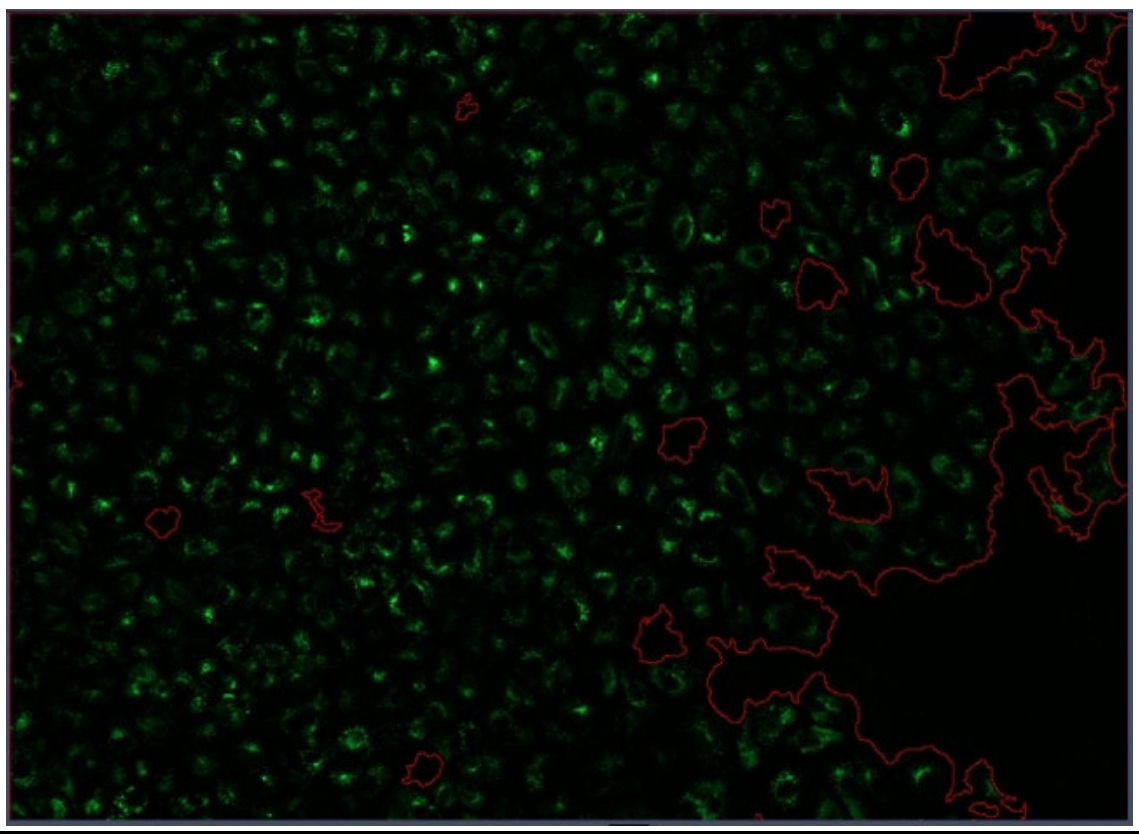

J

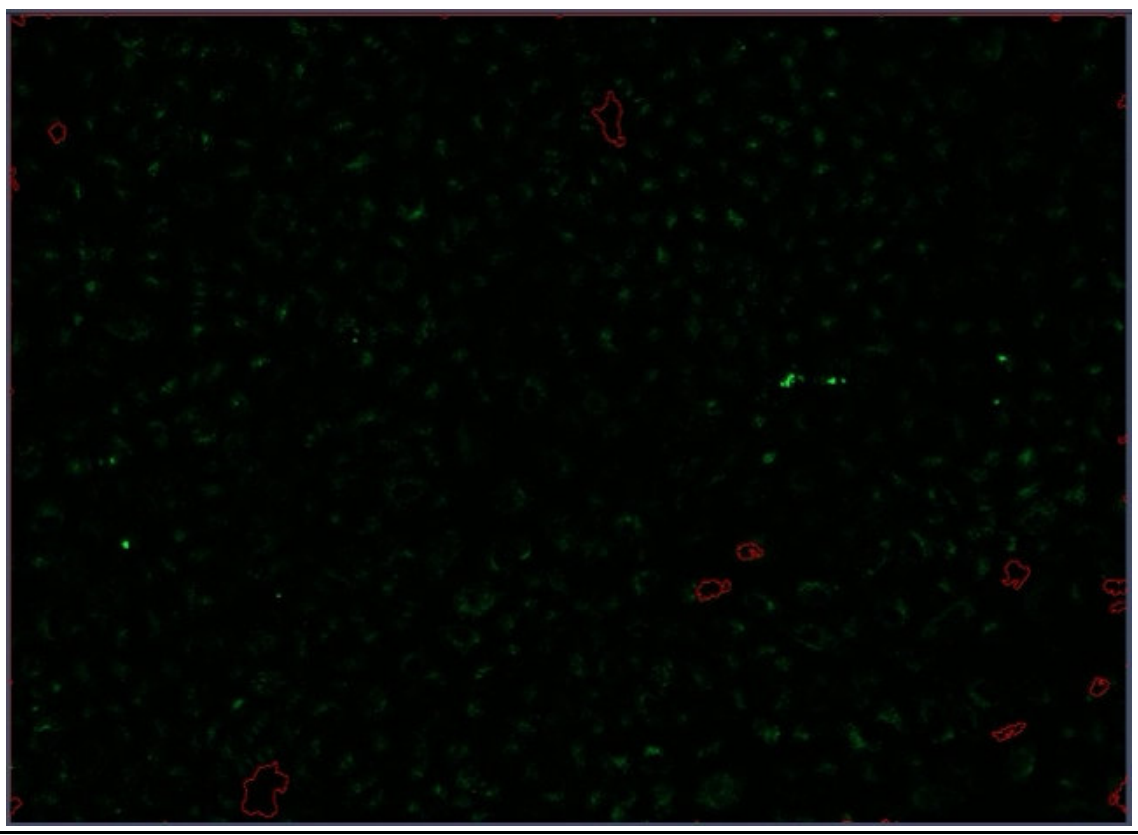

Fig. S2: Representative images of the segmentation in image analysis of two different biological replicates of A549 cells after incubation with 31 µg·cm^{-2} fluorescently labeled PS particles for 24 h. A/B) Images of all channels merged. C/D) Overlay image of Hoechst 33342 filter image with the PS-NPs filter image and the outlining for the Hoechst filter at 2200 r.f.u. E/F) Overlay image of Hoechst 33342 filter image with the PS-NPs filter image and the outlining for the Hoechst filter at 2500 r.f.u. G/H) Overlay image of the outlining obtained from 1100 r.f.u. in the Alexa Fluor 647 Phalloidin filter image on the whole image for the filtersetting for the fluorescentily labeled PS-NPs. I/J) Overlay image of the outlining obtained from 1300 r.f.u. in the Alexa Fluor 647 Phalloidin filter image on the whole image for the filtersetting for the fluorescentily labeled PS-NPs.

S3 Calculation of the average polymer mass per PS-NP assuming low polydispersity

$$Volume\ (Sphere) = \frac{4}{3}\pi \cdot r^3$$

$$r\ =\ radius\ =\ \frac{diameter}{2} = \frac{180\ nm}{2} = 90\ nm$$

$$Volume\ (Sphere) = \frac{4}{3} \cdot \pi \cdot 90\ nm^{\ 3} = 3,053,628\ nm^3 = 0.003053628\ \mu m^3$$

$$Density(Polystyrene) = 1.09\ \frac{g}{cm^3} = 1.09\frac{pg}{\mu m^3}$$

$$Mass(Sphere) = Volume(Sphere) \cdot Density(Polystyrene)$$
$$=\ 1.09\frac{pg}{\mu m^3} \cdot 0.003053628\ \mu m^3\ = 0.00333\ pg = 3.33\ fg$$

S4 Py-GC-MS chromatograms and mass spectra of styrene trimer and 4F-styrene trimer

A)

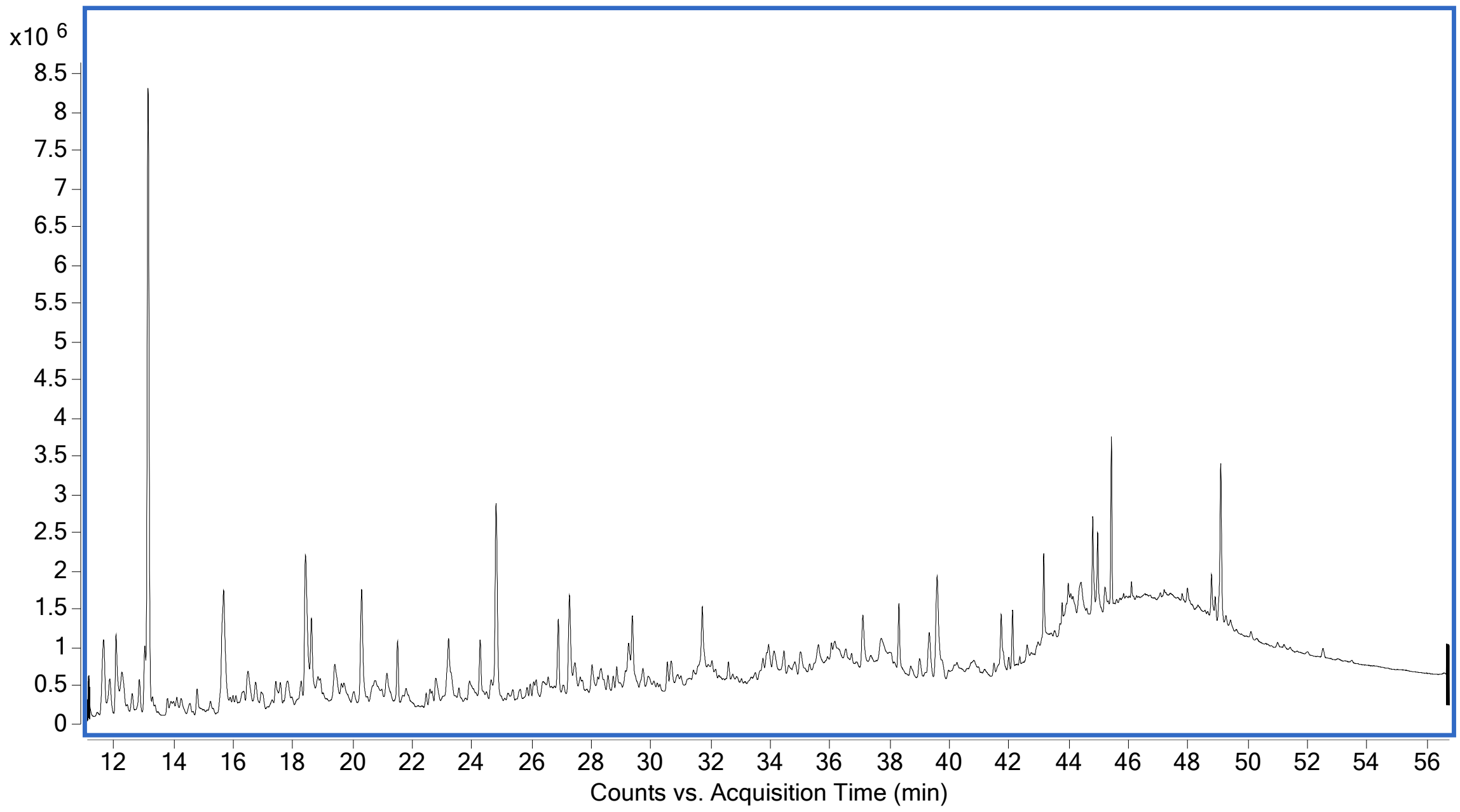

B)

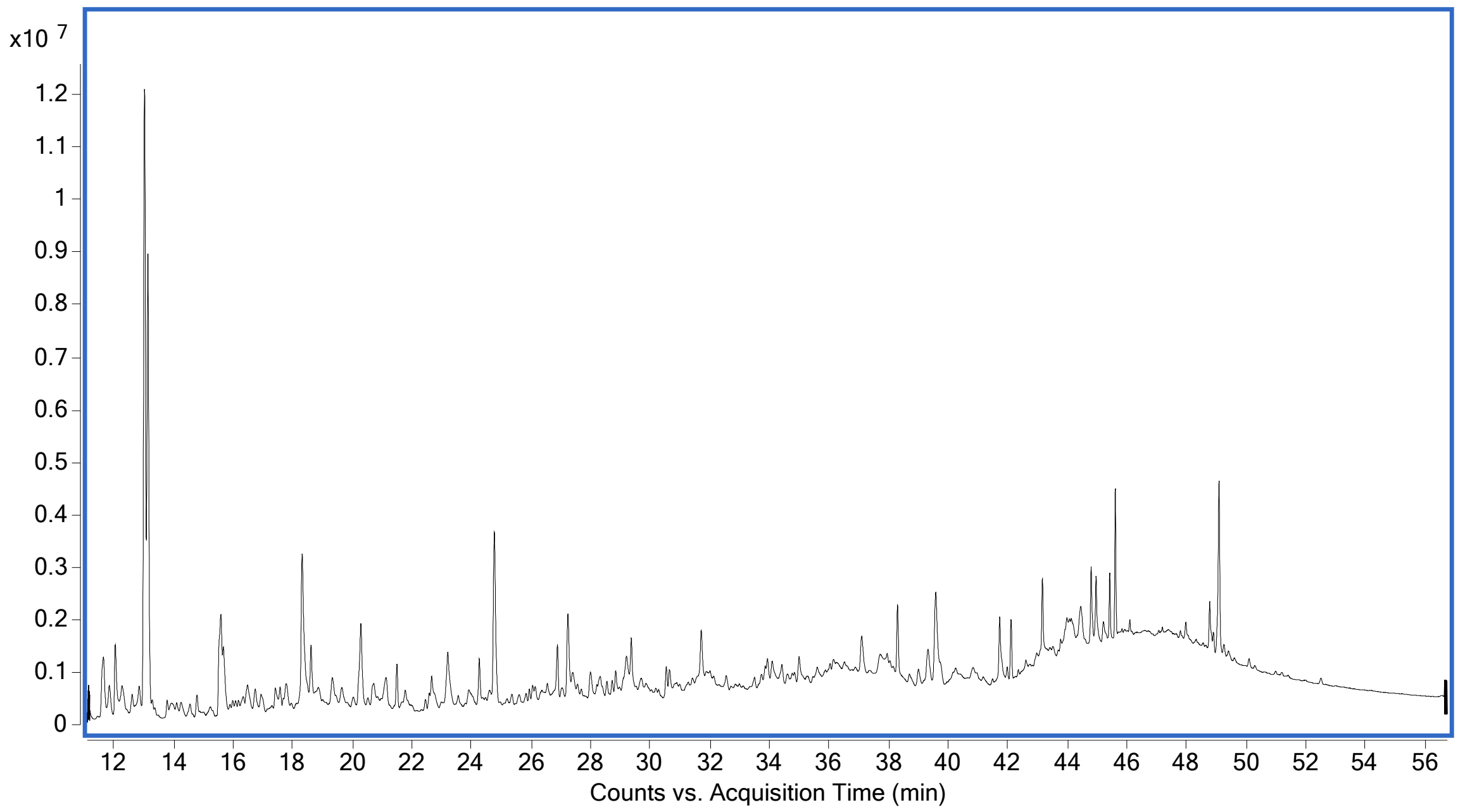

C)

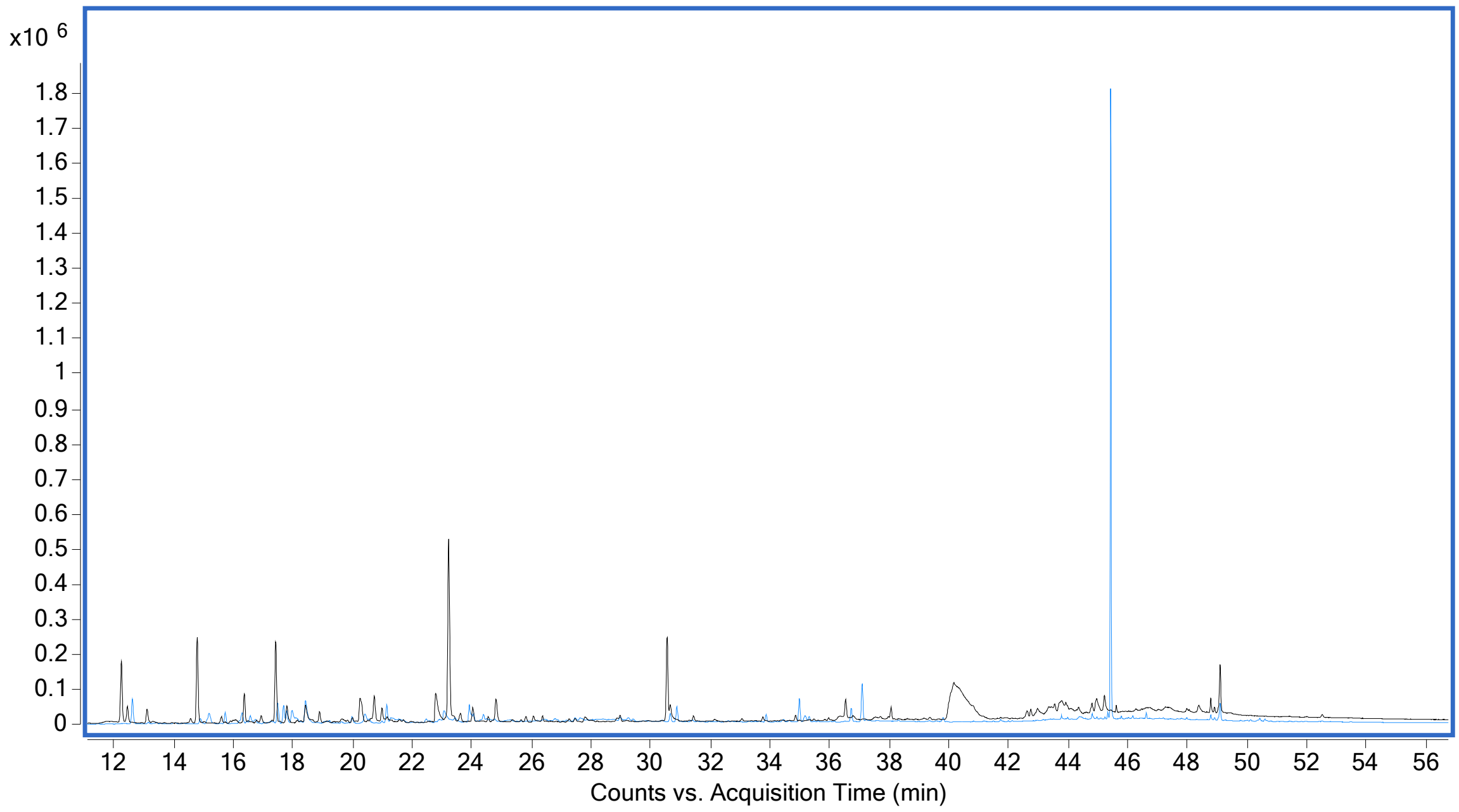
x10 6
1.8
1.7
1.6
1.5
1.4
1.3
1.2
1.1
1
0.9
0.8
0.7
0.6
0.5
0.4
0.3
0.2
0.1
0
12 14 16 18 20 22 24 26 28 30 32 34 36 38 40 42 44 46 48 50 52 54 56
Counts vs. Acquisition Time (min)

D)

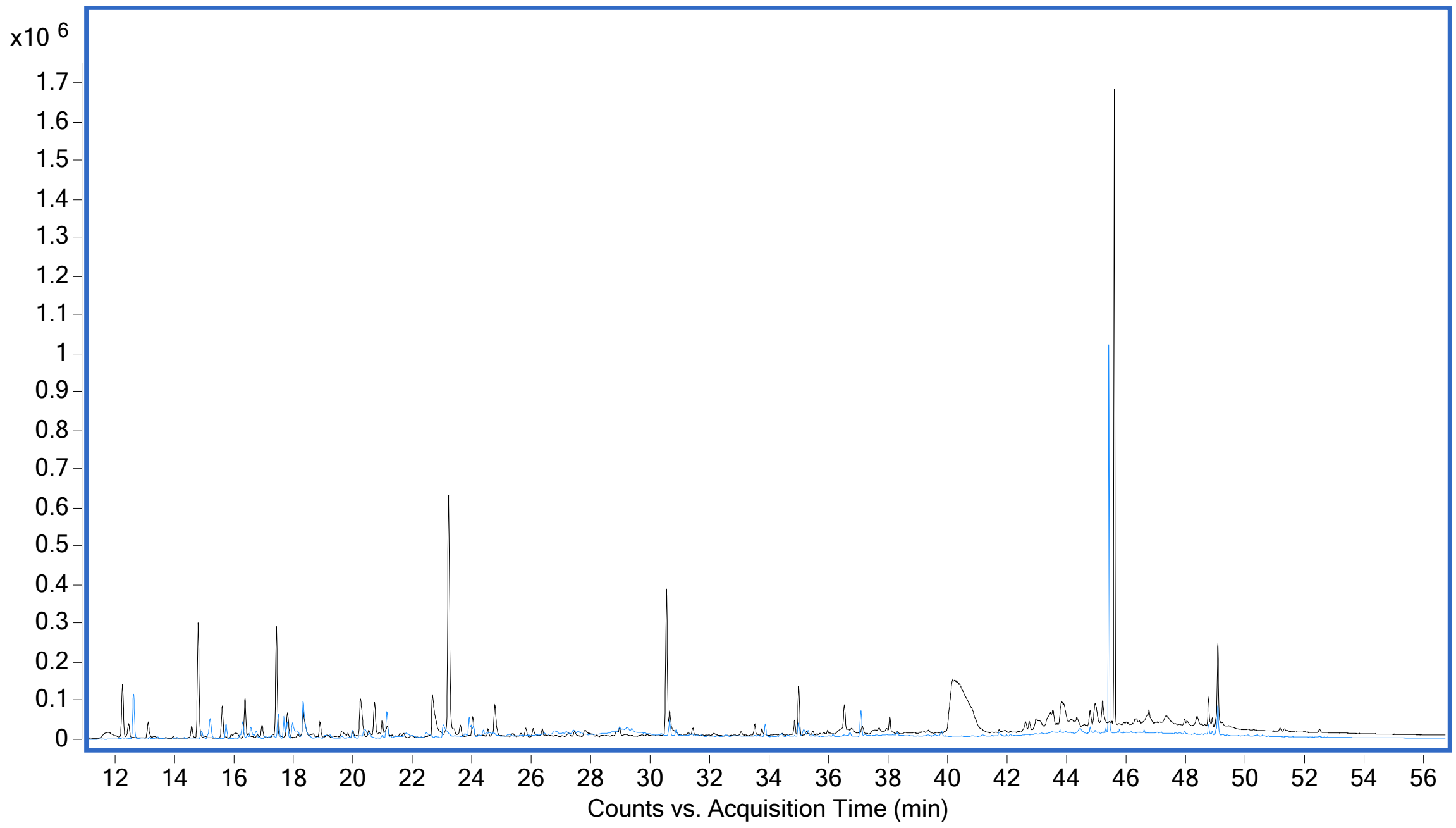
x10 6
1.7
1.6
1.5
1.4
1.3
1.2
1.1
1
0.9
0.8
0.7
0.6
0.5
0.4
0.3
0.2
0.1
0
12 14 16 18 20 22 24 26 28 30 32 34 36 38 40 42 44 46 48 50 52 54 56
Counts vs. Acquisition Time (min)

E)

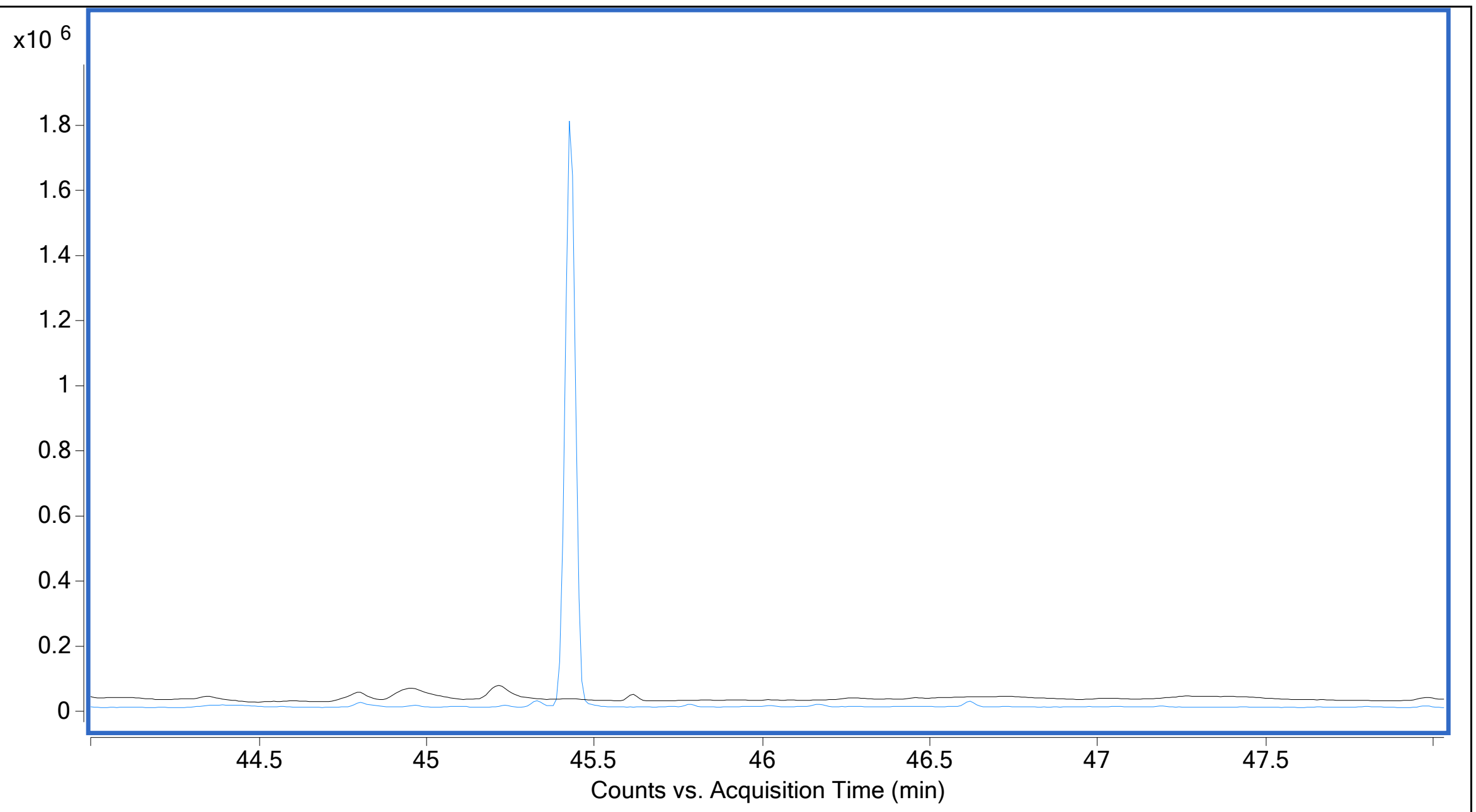
x10 6
1.8
1.6
1.4
1.2
1
0.8
0.6
0.4
0.2
0
44.5
45
45.5
46
46.5
47
47.5
Counts vs. Acquisition Time (min)

F)

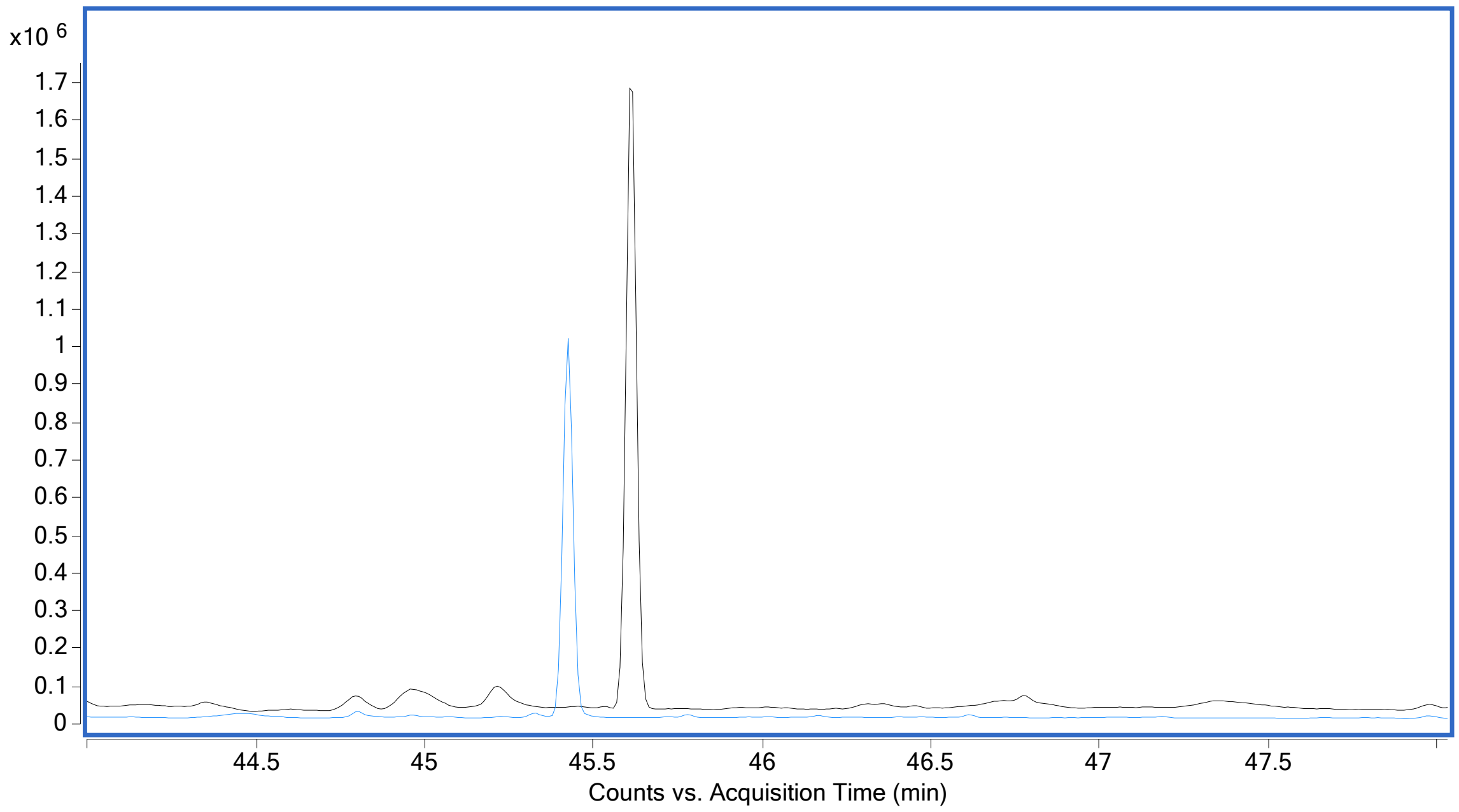
x10 6
1.7
1.6
1.5
1.4
1.3
1.2
1.1
1
0.9
0.8
0.7
0.6
0.5
0.4
0.3
0.2
0.1
0
44.5
45
45.5
46
46.5
47
47.5
Counts vs. Acquisition Time (min)

G)

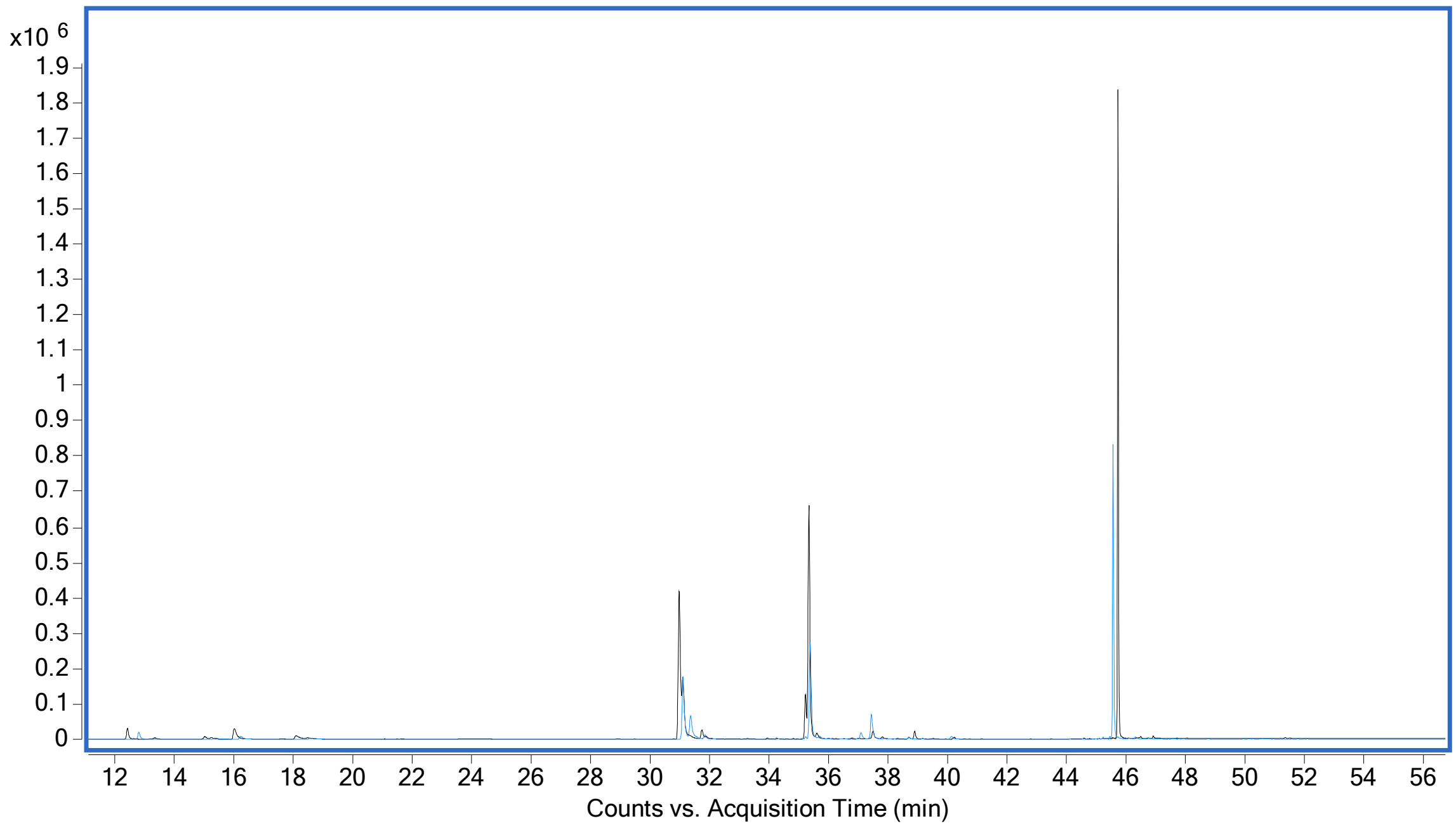
x10 6
1.9
1.8
1.7
1.6
1.5
1.4
1.3
1.2
1.1
1
0.9
0.8
0.7
0.6
0.5
0.4
0.3
0.2
0.1
0
12 14 16 18 20 22 24 26 28 30 32 34 36 38 40 42 44 46 48 50 52 54 56
Counts vs. Acquisition Time (min)

H)

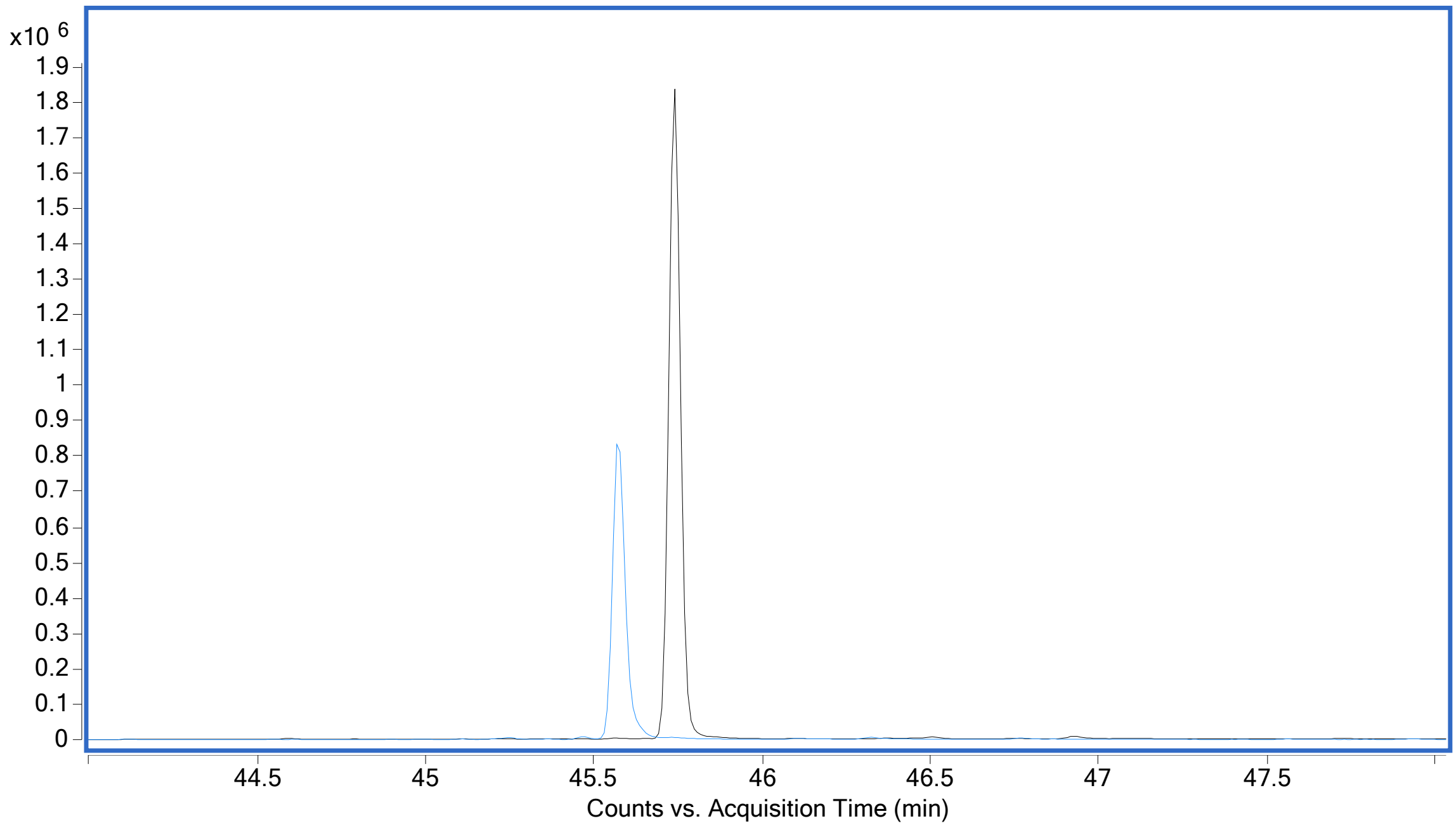
x10 6
1.9
1.8
1.7
1.6
1.5
1.4
1.3
1.2
1.1
1
0.9
0.8
0.7
0.6
0.5
0.4
0.3
0.2
0.1
0
44.5 45 45.5 46 46.5 47 47.5
Counts vs. Acquisition Time (min)

I)

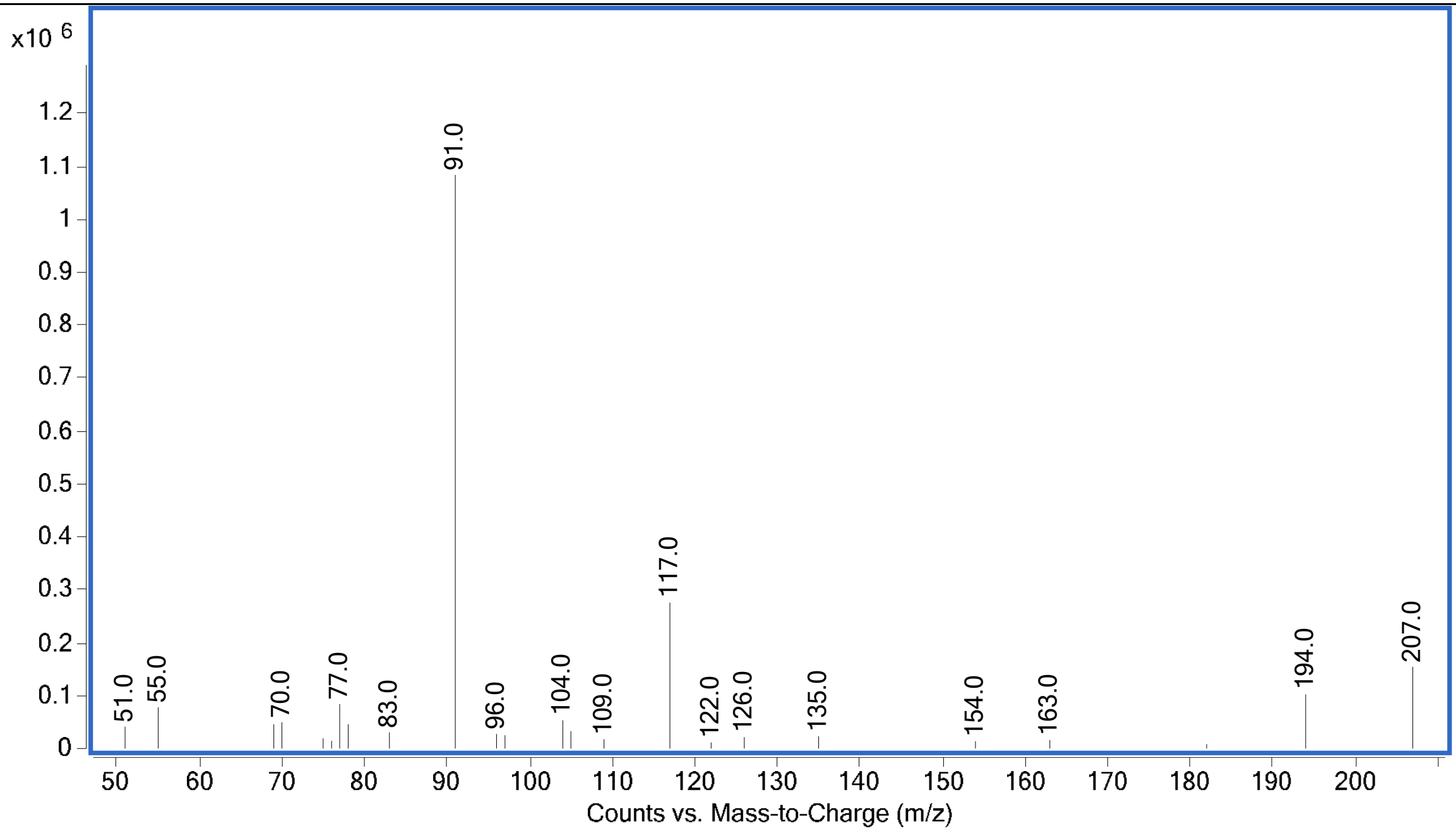

J)

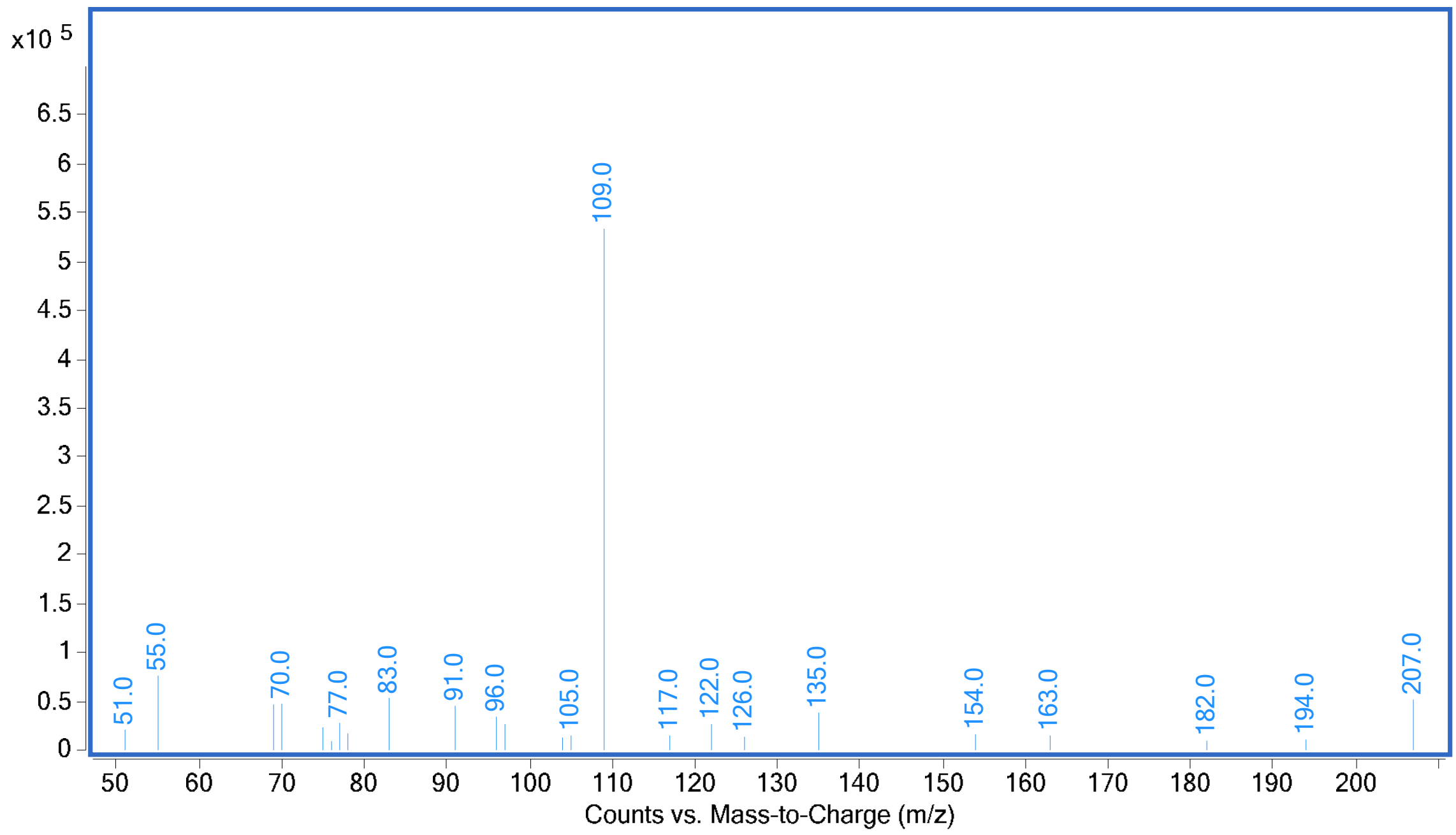

Fig. S4: Representative total ion chromatograms (TIC) of A) blank samples (A549 cells not incubated with PS-NPs) and B) A549 cells incubated with the highest tested dose of 31 µg·cm^{-2} PS-NPs. Representative extracted ion chromatograms (EIC) of the PS-trimer (*m/z* = 91, black) and the 4F-PS-trimer (*m/z* = 109, blue) of C) blank samples and D) A549 cells incubated with the highest tested dose of PS-NPs.

EICs for t_R 44–48 min depicting the peaks used for quantification of E) the blank sample shown in C) and F) the sample incubated with the highest dose of PS-NPs shown in D).

G) Representative EICs of the PS-trimer (m/z = 91, black) and the 4F-PS-trimer (m/z = 109, blue) for the highest calibration point. H) EICs for t_R 44–48 min depicting the peaks used for quantification in G).

Mass spectra of I) the styrene trimer at t_R = 45.6 min and J) the 4F-styrene trimer at t_R = 46.8 min for the highest calibration point.

S5 Representative Images for automated fluorescence imaging microscopy

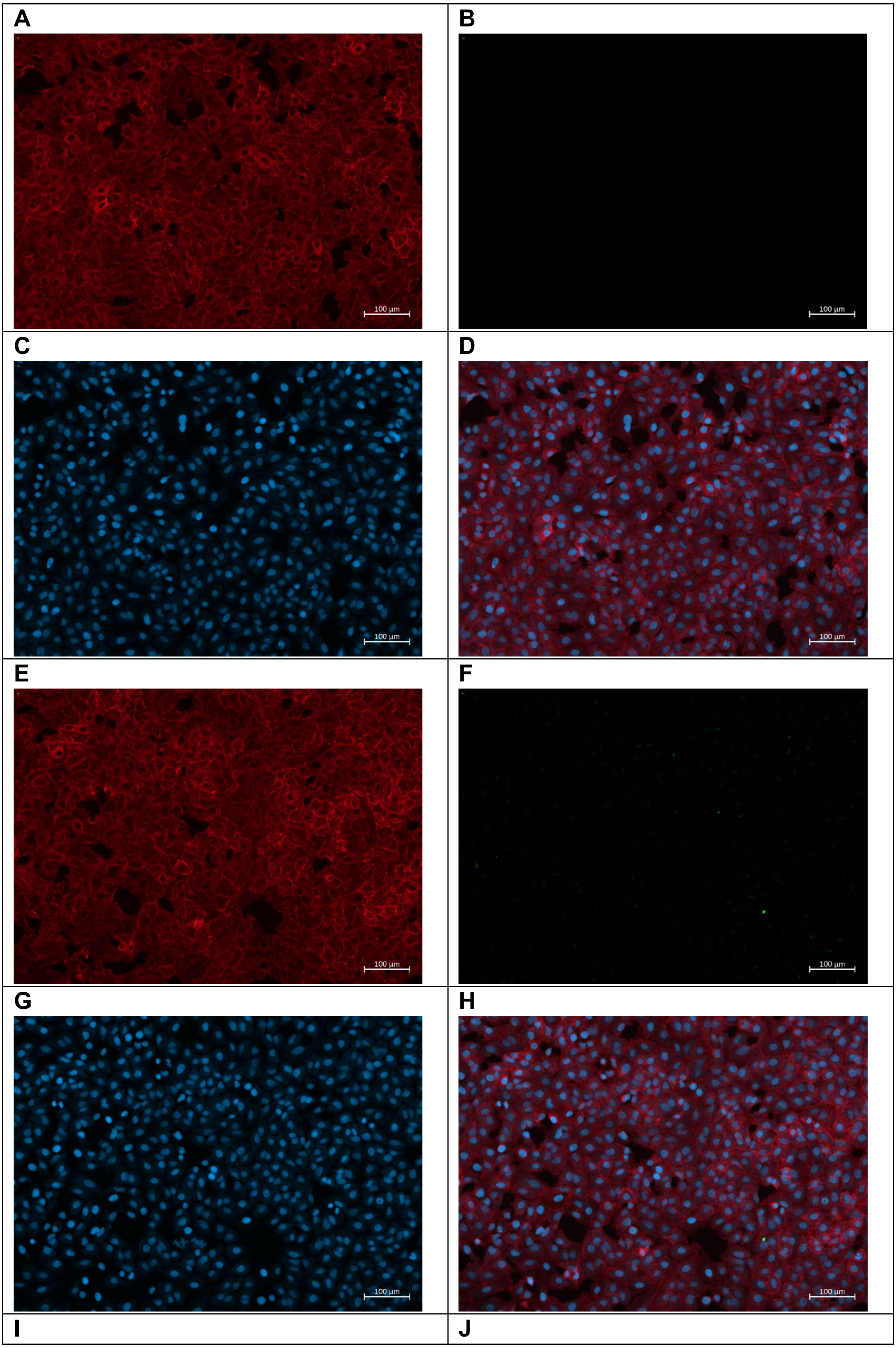

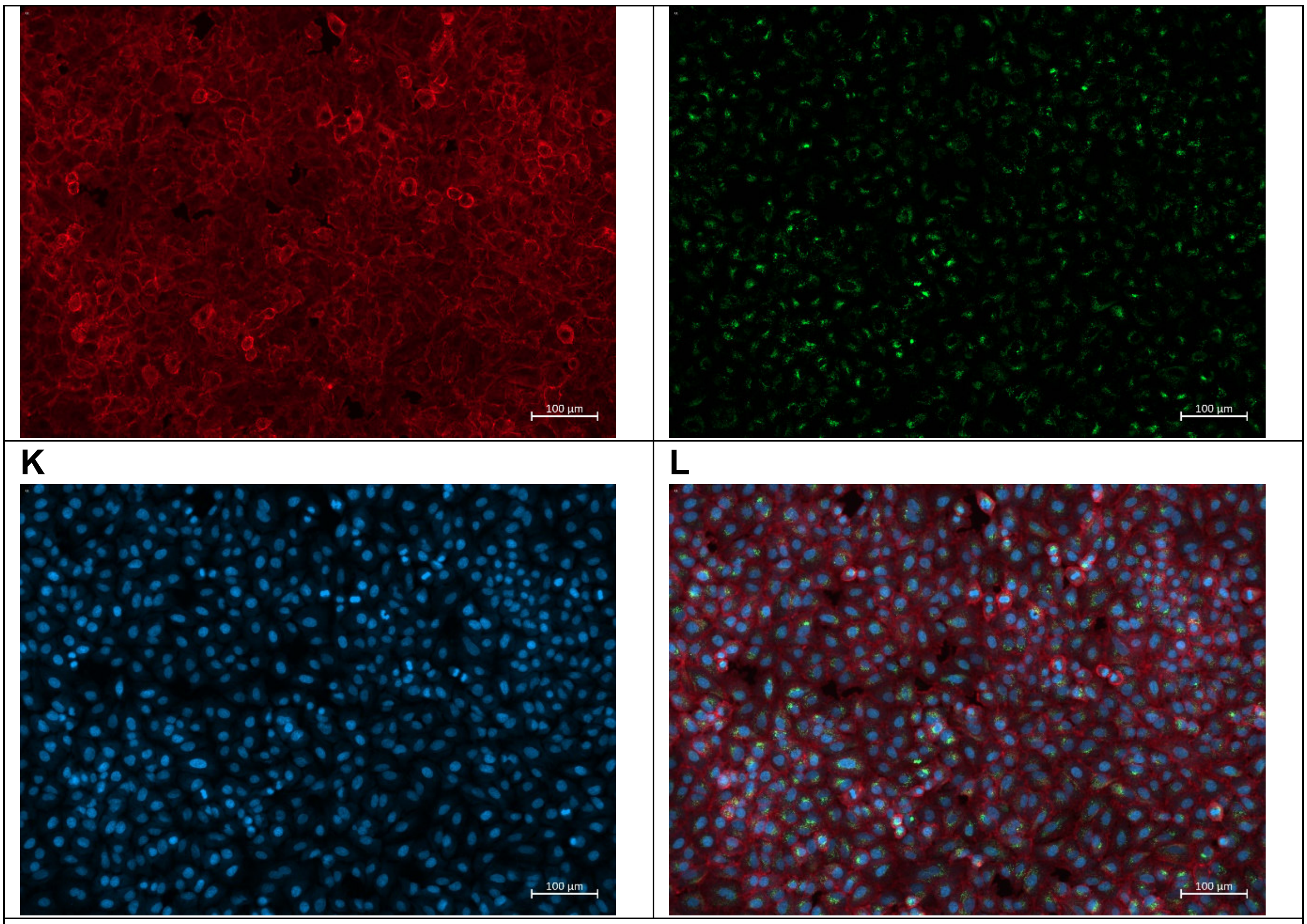

Fig. S5: Representative Images of A549 cells incubated with different concentrations of PS nanoparticles for 24 h. A-D) Images of the negative control for the filter set for Alexa Fluor 647 Phalloidin (A), fluorescently labeled PS-NPs (B), Hoechst 33342 (C) and all three channels merged (D). E-H) Images of A549 cells incubated with 7.8 µg·cm^{-2} fluorescently labeled PS particles for the filter set for Alexa Fluor 647 Phalloidin (E), fluorescently labeled PS-NPs (F), Hoechst 33342 (G) and all three channels merged (H). I-L) Images of A549 cells incubated with 31 µg·cm^{-2} fluorescently labeled PS particles for the filter set for Alexa Fluor 647 Phalloidin (I), fluorescently labeled PS-NPs (J), Hoechst 33342 (K) and all three channels merged (L).

S6 Comparison of uptake of A549 with and without formaldehyde fixation prior to incubation with PS-NPs

To assess potential influence of membrane-adhering particles in automated fluorescence imaging microscopy we tested uptake on non-viable fixed cells. Therefore, cells were seeded as described in Section 2.6 Prior to incubation with particles, cells were washed once with PBS, incubated with 4% formaldehyde solution for 20 min at 37°C and subsequently washed twice with PBS. Incubation with PS-NPs, staining and analysis were performed as described in Section 2.6 A second plate was incubated under standard conditions (“non-fixed”).

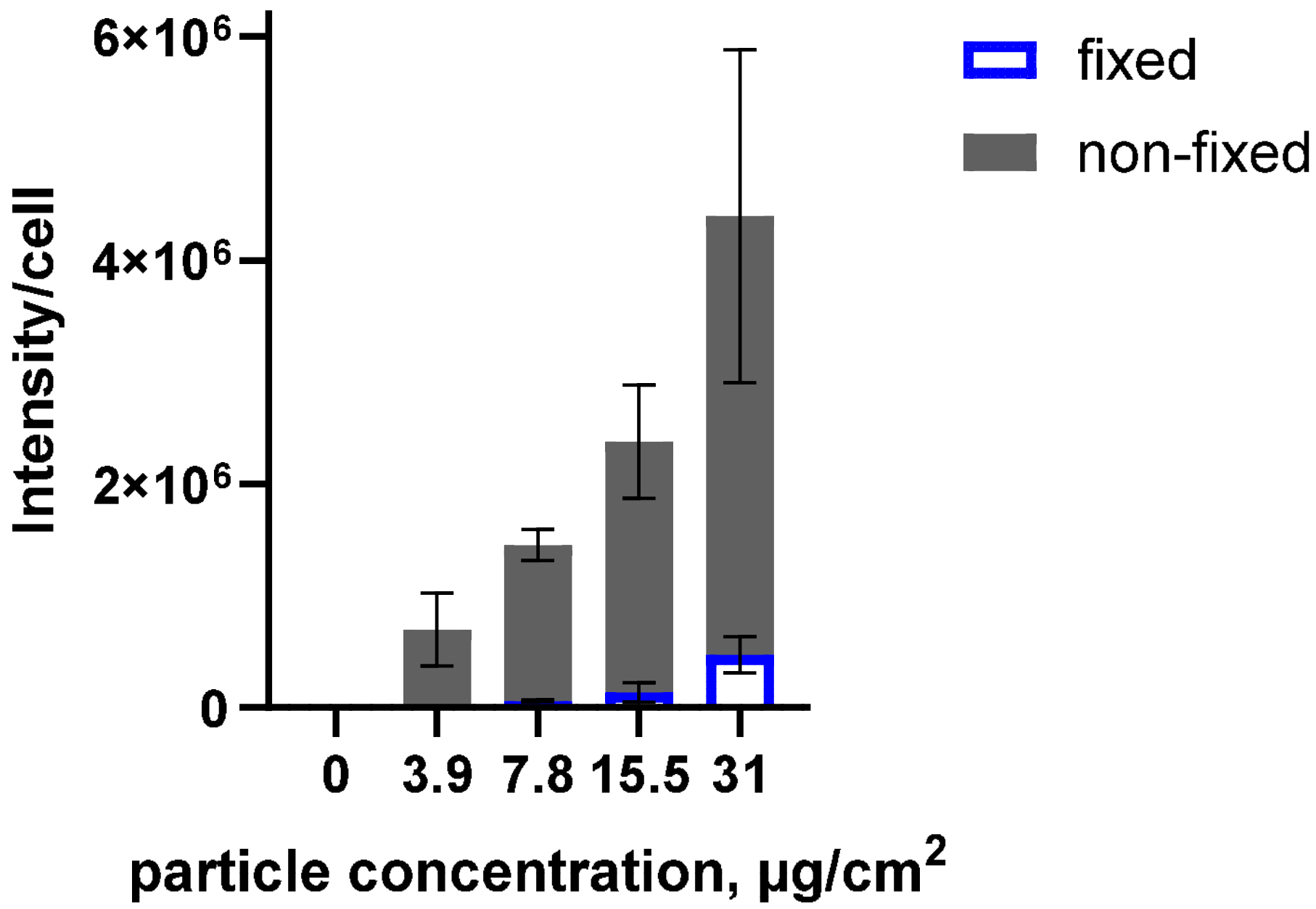

Fig. S6: Average fluorescence intensity of the fluorophore associated with the PS-NPs per cell after exposing A549 cells with increasing concentrations of PS-NPs for 24 h. Fixed cells (blue) underwent fixation with formaldehyde prior to PS-NPs incubation. Non-fixed cells (grey) were incubated 24h after seeding without fixation. Error bars indicate the standard deviation of three technical replicates.

S7 Investigation of potential effects of trypsin incubation on surface adhering particles

To assess the possible influence of trypsin on the adherence of particles to the well plate surface, fluorescently labeled PS-NPs were added at a dose of 31 µg·cm^{-2} in 100 µL cell culture medium to six wells of a 96 well plate and incubated for 24 h. Afterwards, the wells were washed 3 times with PBS and half of the wells were incubated with 0.5% trypsin/0.02% EDTA in PBS. The trypsin was aspirated and all treated wells were filled with PBS before measuring the fluorescence intensity in a plate reader ($\lambda_{ex./em.}$ 490 nm / 520 nm). Non-treated wells were filled with 100 µL PBS and analyzed for autofluorescence, which was subtracted from the results obtained from the treated wells. and the dispersion medium (PBS). The relatively low fluorescence of the non-incubated PBS wells, amounting to about half the signal of the particle-incubated wells (SD = 2.2), indicates an adequate signal-to-background ratio and thus supports the analytical reliability of the measurements.

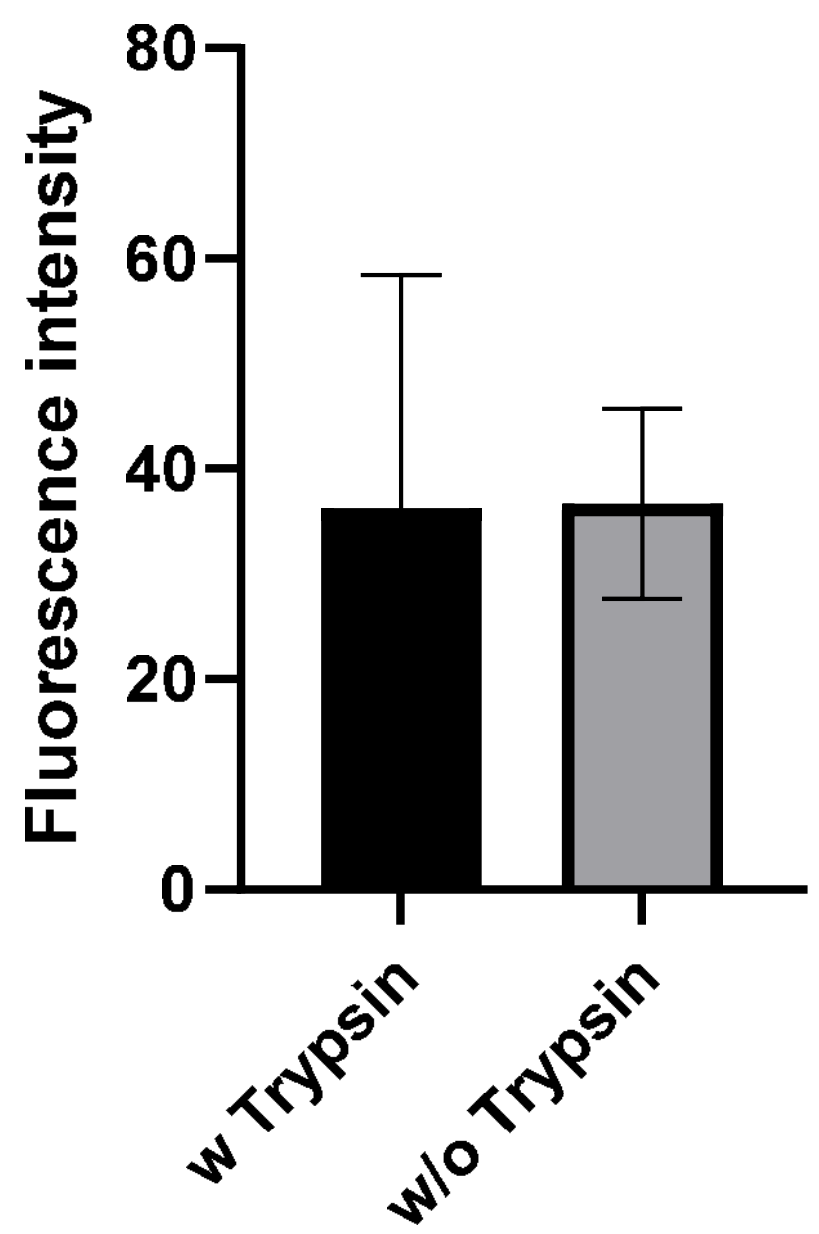

Fig. S7: Fluorescence intensity of the fluorophore associated with the PS-NPs after exposing empty wells with 31 µg·cm^{-2} PS-NPs for 24 h. Each well was washed 3 times with PBS. Trypsinization (left, dark grey) was performed in selected wells for 5 min at 37 °C. Other wells (right, light grey) were meanwhile incubated with PBS for 5 min at 37 °C. Error bars indicate the standard deviation of three technical replicates.

S8 Assessment of the pH dependency of the fluorescence intensity of the fluorescently labeled PS nanoparticles

To asses potential changes in fluorescence intensity due to changes of the pH in different cellular compartments, fluorescently label PS particles were incubated at a dose of 31 µg·cm^{-2} for 10 min in PBS, PBS adjusted to pH 6 or PBS adjusted pH 4 before measuring the fluorescence intensity in a plate reader ($\lambda_{ex./em.}$ 490 nm / 520 nm).

The stable fluorescence measured throughout the tested pH range indicates that the emission of the organic dye incorporated into the PS particles is independent of pH within the physiologically relevant range.

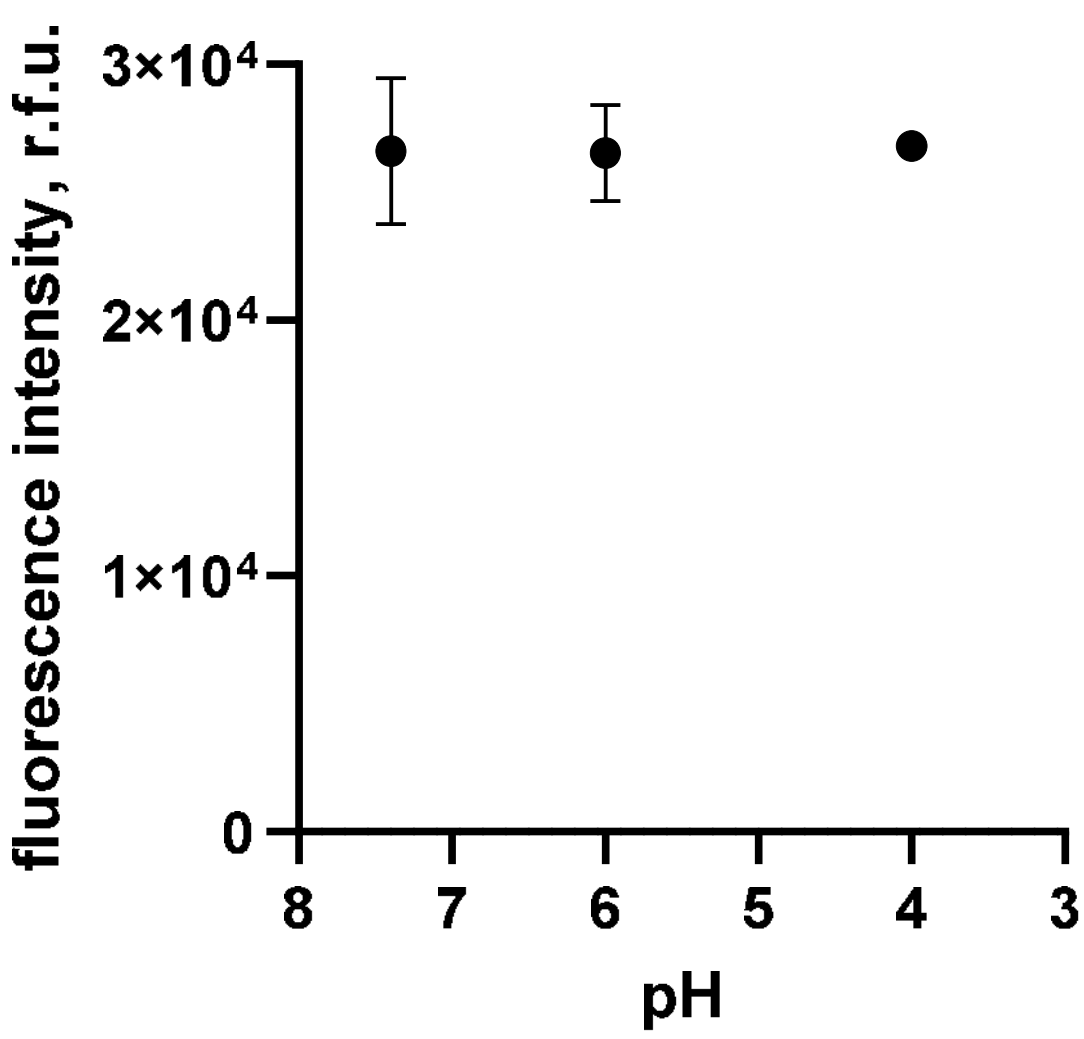

Fig. S8: Fluorescence intensity of the fluorophore associated with the PS-NPs after incubation of 100 µL of 31 µg·cm^{-2} PS-NPs for 10 min at pH 7.4, 6.0 and 4.0. Error bars indicate the standard deviation of three technical replicates.
